# Nonlinear kernel-based fMRI activation detection

**DOI:** 10.3389/fnimg.2025.1649749

**Published:** 2025-09-10

**Authors:** Chendi Han, Zhengshi Yang, Xiaowei Zhuang, Dietmar Cordes

**Affiliations:** ^1^Cleveland Clinic Lou Ruvo Center for Brain Health, Las Vegas, NV, United States; ^2^Department of Psychology and Neuroscience, University of Colorado, Boulder, CO, United States

**Keywords:** data analysis, fMRI, task fMRI, activation, nonlinear kernel, CCA, KCCA

## Abstract

Kernel Canonical Correlation Analysis (KCCA) is an effective method for globally detecting brain activation with reduced computational complexity. However, the current KCCA is limited to linear kernels, and the performance of more general types of kernels remains uncertain. This study aims to expand the current KCCA method to arbitrary nonlinear kernels. Our contributions are twofold: First, we propose an inverse mapping algorithm that works for general types of nonlinear kernels. Second, we demonstrate that nonlinear kernels yield improved performance, particularly when the true neural activation deviates from the hypothesized hemodynamic response function due to the complex nature of neural responses. Our results, based on a simulated fMRI dataset and two task-based fMRI datasets, indicate that nonlinear kernels outperform linear kernels and effectively reduce activation in undesired regions.

## 1 Introduction

In typical fMRI pipelines, univariate general linear models (GLMs) are often applied to spatially smoothed data to improve the signal-to-noise ratio and satisfy the assumptions required for random field theory (Worsley, 1995; Poline et al., 1997; Friman et al., 2003). While this method efficiently reduces noise, it also causes increased spatial blurring. Local canonical correlation analysis (CCA) offers an alternative technique for detecting brain activation and is applicable to a broader range of smoothing filters (Friman et al., 2001; Cordes et al., 2012; Zhuang et al., 2017). Both GLM with Gaussian smoothing and local CCA are categorized as local methods, as they require correlation computation in each local neighborhood around a voxel of interest. In contrast, kernel canonical correlation analysis (KCCA) is a global method that can identify activation in the entire brain in a single step (Hardoon et al., 2004). Previous studies demonstrate that linear KCCA significantly mitigates the effects of spatial blurring (Yang et al., 2018).

**Figure 1:**
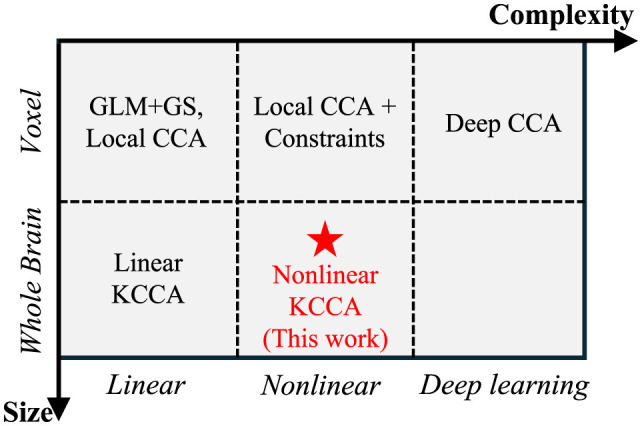
Comparison of different correlation-based activation detection methods, with the horizontal axis representing model complexity and the vertical axis representing data size. While complex models with large data sizes increase the ability to detect signals, they also raise the risk of overfitting. Our work demonstrates an approach that can detect activation globally using nonlinear mapping.

Traditional local CCA and linear KCCA approaches typically assume a linear relationship between the target signal and fMRI data, where the target signal is obtained by convolving the task design with a fixed hemodynamic response function (HRF). However, HRF variability across different subjects has been observed in previous work (Glover, 1999; Lindquist et al., 2009) and is a key consideration when generating simulated data (Erhardt et al., 2012). This variability arises from the complex nature of neuronal responses, making the linear assumption insufficient. Consequently, nonlinear models are necessary to capture the broader range of possible relationships between neural activity and the target signal. Nonlinear relationships can be addressed by transforming data into a nonlinear space prior to applying linear correlation methods (Lai and Fyfe, 2000). Figure 1 provides an overview of all correlation-based activation detection methods, along with their input data sizes and whether they are linear or nonlinear. For example, by imposing constraints, local CCA can learn a variety of relationships between central voxels and their surrounding areas (Zhuang et al., 2017, 2020). Since constrained local CCA cannot be solved analytically, voxel-wise optimization is required to determine activation, which reduces computational efficiency. This limitation highlights the advantages of global methods, such as nonlinear KCCA, for these tasks.

The application of a nonlinear kernel in the KCCA framework is not a novel concept, as kernel methods map the original inputs into the kernel space, which can naturally have high or even infinite dimensionality (Lai and Fyfe, 2000; Melzer et al., 2001; Akaho, 2006). In image-based problems, KCCA has been successfully employed for tasks such as cross-modality retrieval (Hardoon et al., 2004) and identifying relationships between the left and right halves of images (Lopez-Paz et al., 2014; Uurtio et al., 2019). Previous fMRI studies have employed nonlinear kernel-based methods, including support vector machines for classification (Zhang et al., 2014), detecting connectivity (Karanikolas et al., 2016), and predicting fMRI patterns (Ni et al., 2008; Langs et al., 2011). Nonetheless, to the best of our knowledge, nonlinear KCCA has not yet been investigated for fMRI activation analysis.

The primary objective of this study is to extend the current KCCA-based fMRI detection to incorporate nonlinear kernels, thereby enabling the identification of generalized relationships while maintaining computational efficiency. Because the inverse mapping from nonlinear kernels cannot be directly defined (Yang et al., 2018), a key question arises: if a correlation is identified in the kernel space, how can it be back-mapped to obtain the corresponding activation pattern in voxel space? In computer vision, the concept of a heat map is commonly used to measure the importance of each pixel to the final output. Mathematically, this importance is represented by the derivative of the output with respect to the input. However, the derivatives computed from highly nonlinear convolutional neural networks can be noisy, prompting several modifications for specific tasks. For example, Class Activation Maps (CAM) are employed to compute class-discriminative features (Zhou et al., 2016; Selvaraju et al., 2017), while similar techniques, such as sensitivity maps (Smilkov et al., 2017) and saliency maps (Simonyan et al., 2013), evaluate the importance of each pixel for a given problem. These approaches have also been adapted for fMRI studies. In classification-based problems, sensitivity maps are defined as the derivatives of the output with respect to the data, applicable to linear, Gaussian, and parabolic kernels (Rasmussen et al., 2011). In this study, we extend this concept to nonlinear KCCA by computing the derivative from the kernel space to the original space. We demonstrate that this new approach is robust and effective across various kernels and datasets. The proposed nonlinear KCCA method involves several key steps. Following data preprocessing, the fMRI data are mapped to the kernel space using either a linear or nonlinear function. We then derive voxel-defined activation patterns using back-reconstruction algorithms. Finally, we employ different criteria to evaluate the overall performance of the method.

The second question we address is under what conditions nonlinear kernels outperform linear kernels in fMRI activation detection. Although nonlinear kernels and sensitivity maps have been applied in classification-based fMRI studies (Rasmussen et al., 2011), a detailed discussion of their advantages remains absent. An intuitive explanation is that neuronal responses are inherently complex and may not align precisely with the effective design signal. To investigate this, we conducted a control variable analysis on a two-dimensional (2D) model, revealing that the deviation between the added signal and the effective design signal is the primary factor contributing to performance differences. In contrast, signal strength and noise type were found to be less significant. To further evaluate the accuracy of the nonlinear KCCA method, we tested it on three distinct tasks: simulated fMRI data and two real fMRI datasets. For each task, we present group-level accuracy based on specific metrics. Our results indicate that commonly used kernels, such as Gaussian or parabolic kernels, do not significantly improve kernel-based fMRI activation detection. In contrast, bounded kernels, such as the hyperbolic tangent kernel, demonstrate superior performance, providing valuable insights into the potential advantages of nonlinear approaches for fMRI analysis.

This article is organized as follows. In Section 2, we outline the detailed steps and data processing pipeline for the proposed nonlinear KCCA method. Section 3 discusses the results obtained from two simulated datasets. In Section 4, we present the results for two real fMRI datasets. Finally, we address the questions raised earlier and conclude the article.

## 2 Methods

### 2.1 fMRI dataset

#### 2.1.1 HCP

Structural and functional MRI data were obtained from the Human Connectome Project (HCP) database (Van Essen et al., 2012), which includes 3T MRI data. We focus on the working memory task fMRI study. A total of 87 males aged 26–30 years were selected. The fMRI data were acquired over 405 timeframes using a multiband factor of 8, TR/TE = 720/33.1 ms; FA = 52 degrees; 72 slices; spatial resolution = 2 mm × 2 mm × 2 mm and in-plane size = 104 × 90. The first 15 timeframes were removed to avoid an unsaturated T1 signal.

#### 2.1.2 In-house scan

This dataset includes 16 subjects, consisting of eight subjects diagnosed with amnestic mild cognitive impairment (aMCI) and eight cognitively normal controls (NC). Data acquisition was conducted with Institutional Review Board approval using a 3T GE HDx MRI scanner equipped with an 8-channel head coil (Jin et al., 2012). The subjects in both groups were matched for age, education, and right-handedness. The acquisition parameters for the echo-planar imaging (EPI) sequence were: TR/TE = 2,000 ms/30 ms, parallel imaging factor = 2, slices = 25 (coronal oblique, perpendicular to the long axis of the hippocampus), slice thickness/gap = 4.0 mm/1.0 mm, 288 time frames (total scan duration 9.6 min), in-plane resolution 96 × 96 interpolated to 128 × 128, yielding a voxel size of 1.72 × 1.72 × 5 mm^3^. The first 10 s (five timeframes) were removed to avoid an unsaturated T1 signal. High-resolution structural images were also acquired including a standard T1-weighted image (0.43 × 0.43 × 1 mm^3^) and a coplanar standard T2-weighted image (0.43 × 0.43 × 2.5 mm^3^).

#### 2.1.3 fMRI preprocessing

The fMRI data, including both resting state and task fMRI, were minimally preprocessed using the SPM12 package (Ashburner, 2009), including realignment, slice-timing correction, coregistration, and normalization to the MNI atlas (Glasser et al., 2013). A high-pass filter with a cutoff frequency of 1/120 Hz was then applied to remove temporal drift, as recommended by fMRI preprocessing software such as SPM or fMRIPrep (Frackowiak et al., 2004; Esteban et al., 2019). Although realignment (which internally performs motion correction) was applied, motion parameters were not used as regressor (Behzadi et al., 2007), because the physical constraint (head padding) resulted in a maximum head translation and rotation of < 0.7 mm at the cortex, and we aimed to avoid introducing high-frequency artifacts (Chen J. E. et al., 2017; Yakupov et al., 2017). No spatial smoothing was performed. Finally, the data were normalized to have a temporal mean of 0 and a variance of 1. We represent the preprocessed task fMRI data as **Y**∈ℜ^*T*×*Q*^, where *T* denotes the length of time and *Q* represents the number of voxels.

#### 2.1.4 Effective design signal

The HCP working memory task includes three different event types: targets, non-targets, and lures. Each is encoded as a binary time series, which is then convolved with a canonical hemodynamic response function (HRF) to generate a design signal represented as **X**∈ℜ^390 × 3^. For in-house scans, the stimuli consist of four different events: instruction, control, encoding, and recognition. After applying the same HRF convolution to model these four events, the resulting vector is represented as **X**∈ℜ^283 × 4^.

For a specific contrast **C**, the effective design signal **X**_eff_ is generated according to the method described in Cordes et al. (2012)


(1) $$\begin{array}{l} {\text{X}}_{\text{eff}}=\text{X}{\left({\text{X}}^{T}\text{X}\right)}^{-1}\text{C}{\left[{\text{C}}^{T}{\left({\text{X}}^{T}\text{X}\right)}^{-1}\text{C}\right]}^{-1}. \end{array}$$

Next, we map **X**_eff_ into the linear kernel space using the equation $K_{\text{X}}={\text{X}}_{\text{eff}}{\text{X}}_{\text{eff}}^{T}$. Subsequently, we will focus on the effective design signal.

#### 2.1.5 Steerable filters

**Figure 2:**
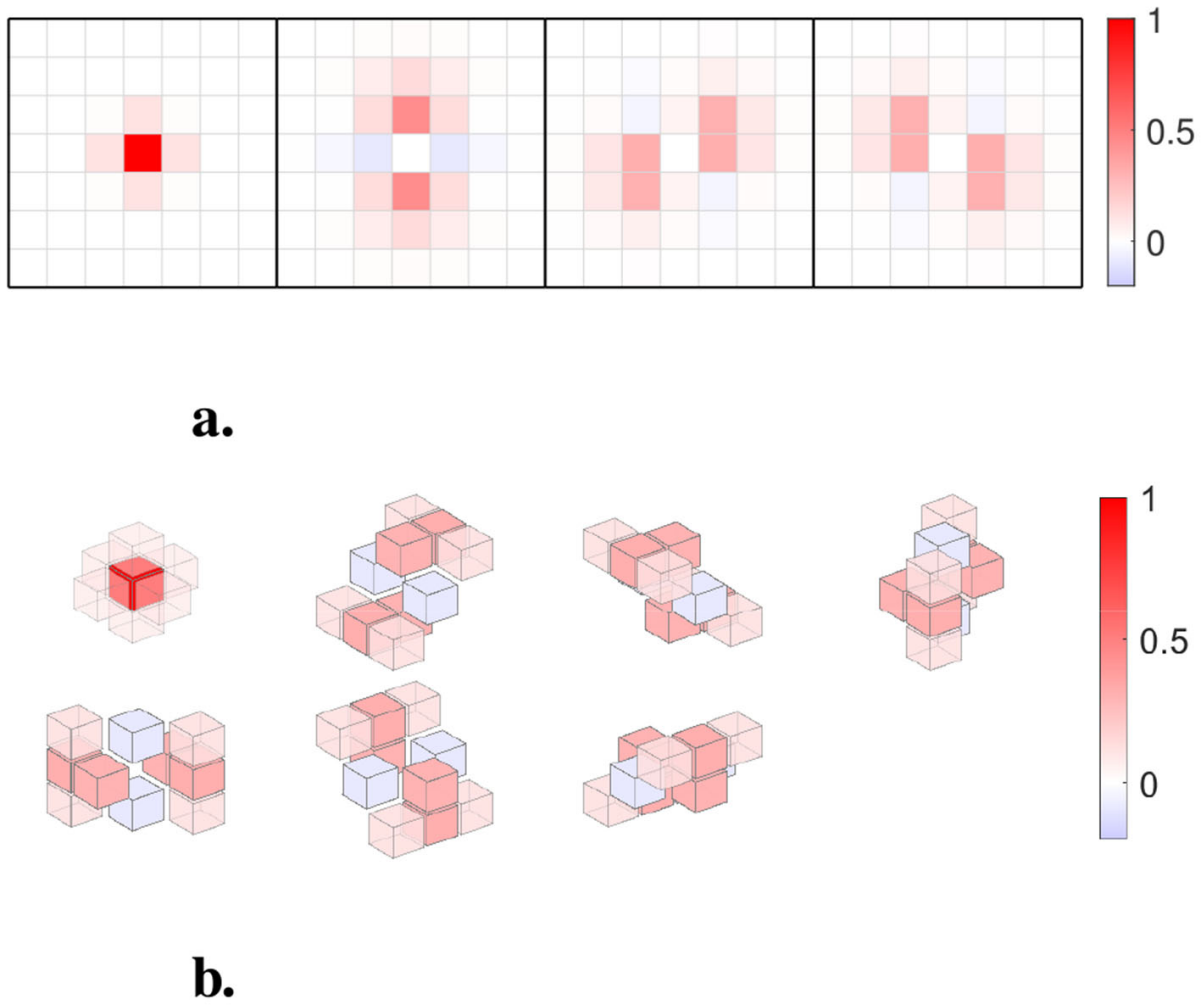
**(a)** Four 7 × 7 Steerable Filters (SF) used for 2D. The equal weight sum equals a single Gaussian filter with FWHM = 2 pixels. **(b)** Seven 7 × 7 × 7 SF are used for 3D fMRI data. The equal weight sum equals a single Gaussian smoothing filter with FWHM = 4mm.

To reduce spatial blurring, we use Steerable Filters (SF) for all the kernel-related problems. The number of SF used depends on the dimension of the data. For the 2D problem, we use four filters (see Figure 2a). The equally weighted summation of these four filters is equivalent to a single full-width at-half-maximum (FWHM) equal to a 2 pixel Gaussian filter. For the 3D problem, we use seven SF generated from a Gaussian kernel with FWHM 4 mm (Yang et al., 2018) (see Figure 2b). The equally weighted summation of these seven filters is equivalent to a single FWHM = 4 mm Gaussian filter. A detailed description of the SF is provided in Appendix 7.1.

**Figure 3:**
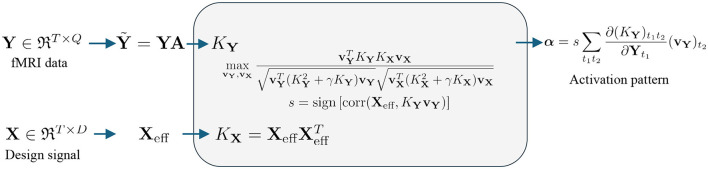
Flow chart for nonlinear kernel-based fMRI activation detection: The fMRI data (upper row) are mapped into kernel space using the kernels listed in Table 1. The design signal (lower row) is mapped into kernel space using a linear kernel. Correlation is computed using KCCA. Once the relationship is established in the kernel space, back-reconstruction algorithms, as defined in Section 2.3, are used to extract the activation pattern.

The flow map for the fMRI signal is shown in Figure 3. To process the fMRI signal, we first perform a linear mapping of the data from **Y** to the feature space $\tilde{\text{Y}}$ using the equation $\tilde{\text{Y}}=\text{Y}\text{A}\in {ℜ}^{T\times P}$, where **A**∈ℜ^*Q*×*P*^ is the spatial transformation matrix, *T* is the number of time points, *Q* is the number of voxels, and *P* is the number of voxels after spatial transformation. For example, standard Gaussian Smoothing (GS) can be performed using a single isotropic Gaussian filter function, resulting in *P* = *Q* (Friman et al., 2003). More recent studies use 7 3D SFs, such that *P* = 7*Q* (Yang et al., 2018). After smoothing, $\tilde{\text{Y}}$ is fed into six different kernels, as shown in Table 1, to map the data from the original space to the kernel space.

**Table 1:** Summary of six kernels.

**Kernels**	**Expression**
Linear	$K_{\text{Y}}\left({\tilde{\text{Y}}}_{i},{\tilde{\text{Y}}}_{j}\right)={\tilde{\text{Y}}}_{i}{\tilde{\text{Y}}}_{j}^{T}$
Parabolic	$K_{\text{Y}}\left({\tilde{\text{Y}}}_{i},{\tilde{\text{Y}}}_{j}\right)={\left({\tilde{\text{Y}}}_{i}{\tilde{\text{Y}}}_{j}^{T}+b^{2}\right)}^{2}$
Gaussian	$K_{\text{Y}}\left({\tilde{\text{Y}}}_{i},{\tilde{\text{Y}}}_{j}\right)=\exp\left(-||{\tilde{\text{Y}}}_{i}-{\tilde{\text{Y}}}_{j}|{|}^{2}/\sigma^{2}\right)$
Inverse	$K_{\text{Y}}\left({\tilde{\text{Y}}}_{i},{\tilde{\text{Y}}}_{j}\right)=1/\sqrt{||{\tilde{\text{Y}}}_{i}-{\tilde{\text{Y}}}_{j}|{|}^{2}+b^{2}}$
Tanh	$K_{\text{Y}}\left({\tilde{\text{Y}}}_{i},{\tilde{\text{Y}}}_{j}\right)=\tanh\left(b{\tilde{\text{Y}}}_{i}{\tilde{\text{Y}}}_{j}^{T}+c\right)$
Mixed Tanh	$K_{\text{Y}}\left({\tilde{\text{Y}}}_{i},{\tilde{\text{Y}}}_{j}\right)=\tanh\left(b_{1}{\tilde{\text{Y}}}_{i}{\tilde{\text{Y}}}_{j}^{T}+b_{2}||{\tilde{\text{Y}}}_{i}-{\tilde{\text{Y}}}_{j}|{|}^{2}+c\right)$

#### 2.1.6 GLM with Gaussian smoothing

We use the general linear model (GLM) with Gaussian smoothing as the baseline model for all kernel methods. The Gaussian filter is selected to achieve the same level of smoothing as SF. In matrix form, **A**∈ℜ^*Q*×*P*^ and *P* = *Q*. The correlation is then evaluated between the smoothed fMRI data and **X**_eff_. Subsequently, we will abbreviate this method as GLM.

### 2.2 Nonlinear kernel canonical correlation analysis

#### 2.2.1 Choice of kernel

Table 1 lists the six kernels used in this study. The traditional linear, Gaussian, and parabolic kernels are based on prior work (Rasmussen et al., 2011). Additionally, we include a kernel from the radial basis function family, known as the inverse square root kernel or inverse multiquadric (Fasshauer, 2007; Salazar et al., 2024). The hyperbolic tangent kernel, abbreviated as Tanh, is commonly used as an activation function in machine learning and is also referred to as the sigmoid kernel. Inspired by mixed kernel analysis (Zhu et al., 2012), we introduced a mixed kernel that combines the traditional linear term with the radial basis function through a linear combination. This kernel, referred to as Mixed Tanh throughout the article, leverages both linear and radial components to improve performance. In the following, we will refer to the radial basis function as quadratic.

As noted in Table 1, all the kernels can be generated using linear and quadratic functions. For the linear and quadratic kernels, the data are normalized to their mean absolute values to ensure consistent data ranges. A detailed formula for this normalization is provided in Appendix 7.3. After nonlinear mapping, the matrix value range can vary, especially for the parabolic or hyperbolic tangent kernels when *b* is small. We perform an additional normalization to maintain consistent data ranges, which improves numerical stability when calculating correlations in the kernel space. Compared to the centralized kernel method (Bengio et al., 2004; Chen B. et al., 2017), tracking derivatives with such normalization is much easier. After normalization, we can demonstrate that the hyperbolic tangent kernel can approximate the linear kernel when *b* is small and *c* = 0, and the mixed hyperbolic tangent kernel can approximate the linear kernel when *b*_1_ is small and *b*_2_ = *c* = 0.

#### 2.2.2 Computing correlation in kernel space

Using KCCA, the eigenvectors **v**_**X**_ and **v**_**Y**_ are determined to maximize the canonical correlation *r* = corr(*K*_**X**_**v**_**X**_, *K*_**Y**_**v**_**Y**_) in the feature space where


(2) $$\begin{array}{l} r=\frac{{\text{v}}_{\text{X}}^{T}K_{\text{X}}K_{\text{Y}}{\text{v}}_{\text{Y}}}{\sqrt{{\text{v}}_{\text{X}}^{T}\left(K_{\text{X}}^{2}+\gamma K_{\text{X}}\right){\text{v}}_{\text{X}}}\sqrt{{\text{v}}_{\text{Y}}^{T}\left(K_{\text{Y}}^{2}+\gamma K_{\text{Y}}\right){\text{v}}_{\text{Y}}}}. \end{array}$$

In this equation, γ is a regularization parameter to avoid overfitting.

### 2.3 Back-reconstruction methods

KCCA maximizes the correlation in the kernel space; however, our goal is to obtain the activation in the original voxel space, represented as α∈ℜ^*Q*×1^. Based on the linear kernel result without smoothing, $\alpha \propto {\text{Y}}^{T}{\text{v}}_{\text{Y}}$ (Hardoon et al., 2004; Yang et al., 2022; Han et al., 2024), we assume that **Y**^*T*^ in the above formula reflects the importance of each voxel in mapping from the original space to the kernel space. This allows us to extend the formula to more general types of nonlinear kernels.

Specifically, let **Y**_*t*_ denote the fMRI data at time *t*, and (_*K*_**Y**_)*t*_1_*t*_2__ denotes the matrix elements indexed by *t*_1_ and *t*_2_. We define α as follows:


(3) $$\begin{array}{l} \alpha =s\underset{t_{1}t_{2}}{\sum }\frac{\partial {\left(K_{\text{Y}}\right)}_{t_{1}t_{2}}}{\partial {\text{Y}}_{t_{1}}}{\left({\text{v}}_{\text{Y}}\right)}_{t_{2}}, \end{array}$$

where *s* = sign[corr(*X*_eff_, *K*_**Y**_**v**_**Y**_)] measures the sign of the correlation between **X**_eff_ and the fMRI signal in kernel space. The whole pipeline is listed in Figure 3.

We offer several comments on this method: First, consider the special case of the linear kernel. As proven in the Appendix 7.3, this method is consistent with a previously published result (up to a constant), regardless of the smoothing filter, specifically $\alpha \propto \text{A}{\text{A}}^{T}{\text{Y}}^{T}{\text{v}}_{\text{Y}}$ (Hardoon et al., 2004; Yang et al., 2022; Han et al., 2024). Second, this method preserves the sign of the activation. This means that α obtained from the BC algorithm can be regarded as the effective correlation by the kernel method, making it generally suitable for a wide range of tasks with activated or deactivated regions. This equivalence is further supported by the similarity of activation patterns observed in later studies, which also validates this algorithm. Third, regarding computational speed, this method can be expressed as a matrix operation with a given **v**_**Y**_, as shown in Appendix 7.4, with a time complexity of *O*(*TQ*). Fourth, we note that a similar idea was used to obtain the activation pattern for classification-based problems. For example, this includes CAM (Zhou et al., 2016; Selvaraju et al., 2017), sensitivity maps (Smilkov et al., 2017), and saliency maps (Simonyan et al., 2013).

### 2.4 Model validation

We propose two different metrics to evaluate the performance of various kernels. For simulated data with known ground truth, we use Metric 1. For real fMRI data with unknown ground truth, we use Metric 2.

#### 2.4.1 Metric 1: ground truth

For the simulated data described in Section 3, where the active voxels are known, we generate a Receiver Operating Characteristic (ROC) curve by setting different thresholds for |α|. The performance is evaluated using the Area Under the Curve (AUC).

In fMRI data analysis, minimizing false positives is crucial. Previous studies commonly used an area for the False Positives Rate (FPR) of < 0.1 (Skudlarski et al., 1999). We define the final accuracy as the ratio between the AUC of the kernel method and GLM:


(4) $$\begin{array}{l} r_{\text{Truth}}=\frac{{\text{AUC(kernel)}}_{\text{FPR}<0.1}}{{\text{AUC(GLM)}}_{\text{FPR}<0.1}}. \end{array}$$

This ratio, *r*_Truth_, was evaluated for both the 2D toy model and the simulated HCP dataset. A value of *r*_Truth_>1 indicates that the kernel method outperforms GLM, whereas *r*_Truth_ < 1 reflects worse performance. The ratio is independent of the number of voxels selected for activation.

#### 2.4.2 Metric 2: activation in gray matter vs. non-gray matter

Real brain activation is more likely to occur in gray matter than in other brain tissues (CSF and white matter) (Gawryluk et al., 2014; Schilling et al., 2023). Following the same idea in Metric 1, we define true positive (activation in gray matter) and false positive (activation not in gray matter) to generate the ROC curve. Gray matter is generated from the SPM package's segmentation with a probability threshold set to >0.5. Note that the number of voxels in GM is significantly larger than the number of activated voxels, which results in the AUC being close to 0.5.

Similarly, we aim to minimize false positives and reduce activation in non-gray matter, particularly in voxels that the method strongly predicts as active. We define the objective ratio for gray matter activation overlay as follows:


(5) $$\begin{array}{l} r_{\text{Gray}}=\frac{{\text{AUC(kernel)}}_{\text{FPR}<0.1}}{{\text{AUC(GLM)}}_{\text{FPR}<0.1}}. \end{array}$$

This ratio, *r*_Gray_, is evaluated for HCP and in-house scans. A larger ratio indicates that the activation is more concentrated in gray matter than in other areas. While this ratio is independent of activation threshold, it is influenced by the threshold for gray matter (GM). We examined the output probabilities from segmentation using the SPM package, which are predominantly concentrated around 1 or 0, resulting in very few voxels within the margin.

### 2.5 Hyperparameter optimization and null distribution

To optimize the hyperparameters in the kernel method, as listed in Table 1, we implement the voxel shuffling algorithm to shuffle the voxels' locations while maintaining their spatial relationships (Zhuang et al., 2017). The optimal hyperparameters are chosen based on the activation that shows the greatest robustness to these data augmentations. The shuffling method is described below:

1. Choose a specific kernel as listed in Table 1, the fMRI data **Y**, and the effective design signal with the given contrast **C**.
2. Starting from a set of hyperparameters, compute the activation map α using the back-reconstruction algorithm proposed in Section 2.3.
3. Rank all voxels based on their α values. Assume that the top 10% of voxels with the highest α (or |α|) values are activated, and the rest are non-activated. Let the number of activated voxels be *Q*_+_, and the number of non-activated voxels be *Q*_Non_.
4. Among the non-activated voxels, rank them by α and choose *Q*_1_ voxels close to the decision boundary. Similarly, for the activated voxels, select *Q*_2_ voxels near the decision boundary.
5. Reverse the voxel order within *Q*_1_ and *Q*_2_ based on their α value. For example, within *Q*_1_, switch the location of the voxel with the largest α and the smallest α, then the second largest and second smallest, and so on. Repeat this process for *Q*_2_.
6. Let the fMRI data after shuffling be denoted as **Y**′. Using the same kernel and hyperparameters, compute the activation α′.
7. Compute the similarity between α and α′. The similarity is defined as follows: treat the top 10% of voxels in α as the ground truth, then apply varying thresholds to α′ to compute the following: true positives—activated voxels that remain activated after shuffling; and false positives—non-activated voxels that become activated after shuffling. A similar AUC curve, called the apparent AUC curve, was proposed in Zhuang et al. (2017). The AUC with FPR < 0.1 is defined as the similarity.
8. In practice, we perform two shuffling procedures and average their results. In the first, set *Q*_1_ = *Q*_2_ = 0.5*Q*_+_; in the second, set *Q*_1_ = *Q*_Non_ and *Q*_2_ = *Q*_+_. The results are combined with equal weights.
9. Repeat steps 2–8 until the hyperparameters with the greatest robustness to voxel shuffling are identified.

The rule is generally applied to all simulated and real fMRI datasets and works for both 2D and 3D simulations.

Since activation values are continuous, we need criteria to determine the activation threshold. A common approach is to resample resting-state data, calculate the correlation, and use this correlation as the null distribution to define FPR (Bullmore et al., 2001; Laird et al., 2004; Nandy and Cordes, 2003).

However, due to kernel mapping, tracking the exact values during the computation pipeline is challenging, especially when the method involves several normalization steps. In this study, we compute the null distribution by shuffling **v**_**Y**_. Specifically, we apply random time shuffling to (_**v**_**Y**_)null_ and then use the randomly shuffled (_**v**_**Y**_)null_ as a constant to compute α_null_. The threshold is then gradually decreased until the false activation rate reaches 0.05, analogous to the commonly used *p* value in previous publications. For GLM, we randomly shuffle **X**_eff_ to compute α_null_.

Compared to the previous studies, this study exhibits a relatively high FPR, because we have not changed the spatial domain, with shuffling performed only in the kernel dimension. Consequently, the computed α_null_ may yield relatively high values, necessitating a higher FPR. We will further discuss this in the Discussion section. Nevertheless, the specific threshold value does not affect the accuracy.

### 2.6 Data summary

Table 2 lists the four datasets and corresponding methods for activation detection and evaluation. Section 3.1 describes a two-dimensional example in which we aim to study the effect of signal strength, noise type, and signal shift. We repeat this process 200 times and compute the statistical average for each setting. Using normalized units, we establish a GLM with FWHM = 2 pixels as the baseline. Finally, we take the absolute value of α and evaluate the final results using the ground truth.

**Table 2:** List of different datasets, smoothing methods and evaluation metrics.

**Name**	**Number of**	**Method**	**Smoothing**	**Absolutely**	**Evaluation**
	**subjects**			**value**	
2D simulation	200	GLM	1GS	Yes	Ground truth
		Kernel	4SFs		
Simulated fMRI	20	GLM	1GS	Yes	Ground truth
		Kernel	7SFs		
HCP	87	GLM	1GS	No	Gray matter
		Kernel	7SFs		
In-house scan	16 × 2 × 2	GLM	1GS	No	Gray matter
		Kernel	7SFs		

Section 3.2 presents a simulated dataset using the HCP dataset, which includes 20 subjects. Resampled resting states are treated as noise, and effective design signals are added to specific brain regions based on segmentation. For three-dimensional data, we utilize seven SFs, with a GLM of FWHM = 4 mm serving as a reference. Performance is evaluated by taking the absolute value of α and comparing it to the ground truth. A similar simulation was performed in Yang et al. (2020).

The dataset discussed in Section 4.1 includes 87 subjects from the HCP dataset, where activation is calculated based on a single contrast. The sign of α is retained, and performance is evaluated based on activation overlaying the gray matter.

In Section 4.2, the kernel method is applied to another real fMRI dataset that includes 16 subjects performing two different tasks with two different contrasts, resulting in 64 realizations. Performance is again evaluated based on activation overlaying in gray matter.

## 3 Simulation

We begin with the simulated data to validate our model's performance. The data was generated using


(6) $$\begin{array}{l} \;Y_{\text{simulated}}=Y_{\text{noise}}+\rho Y_{\text{signal}},\\ {\left(Y_{\text{signal}}\right)}_{q}=\left\{ \begin{array}{l} X_{\text{add}},\text{ for }q\in M\\ 0,\text{ for }q\notin M \end{array}\right. \end{array}$$

where ${\left({\text{Y}}_{\text{signal}}\right)}_{q}\in {ℜ}^{T\times 1}$ is equal to the added signal **X**_add_ if location *q* is activated. **Y**_noise_ is the noise considered independent to **X**, and *M* is a mask that contains all activated voxels. ρ is the signal strength. Numerically, **Y**_noise_ and **X**_add_ are normalized in time with a mean of 0 and a variance of 1.

In Equation 6, the choices for **Y**_noise_, ρ and *M* are task-specific. A consistent feature across all simulations is that *M* includes ~10% of the total number of voxels. Additionally, this study considers scenarios where **X**_add_ and **X**_eff_ are not necessarily equal. In practice, the effective design signal often incorporates several hyperparameters and may not precisely match the true activation signal in the fMRI data. To evaluate model performance more comprehensively, the added signal **X**_add_ is typically designed to have a slight deviation from **X**_eff_ (Yang et al., 2020). We also address this issue in the study and compare the differences between linear and nonlinear kernels.

### 3.1 2D toy model

We begin with a two-dimensional, dimensionless grid of size 100 × 400 to evaluate the model's performance. The main components of this simulation are outlined below.

**Figure 4:**
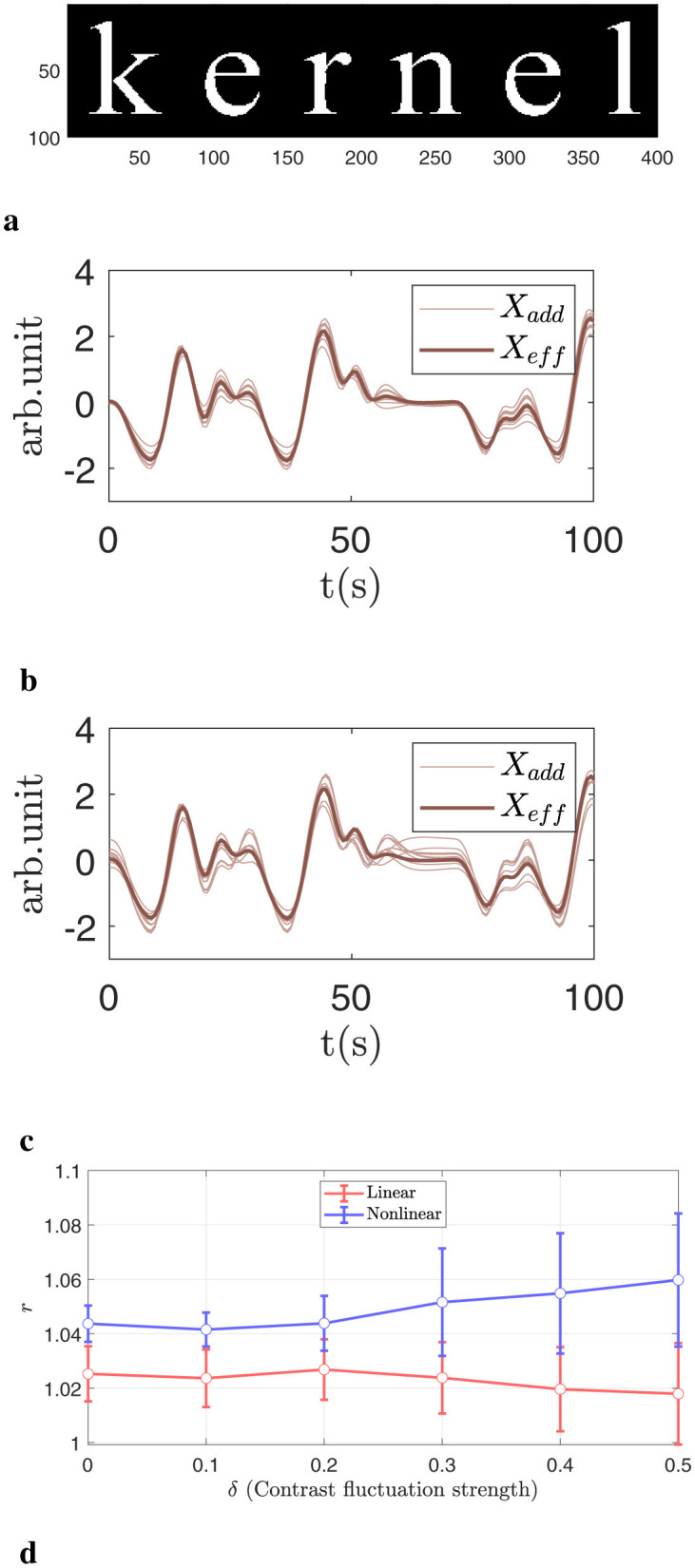
Illustration of the 2D simulation. **(a)** Activation pattern displayed on a 100 × 400 grid. The activated voxels, highlighted in white, form a specific pattern called the “kernel.” **(b)** Comparison of the added signal (thin line) and the effective design signal (thick line) under a small perturbation δ = 0.1. **(c)** Same as **(b)**, but with a larger deviation δ = 0.4. **(d)** Normalized accuracy differences across various δ values. The nonlinear kernel more accurately captures complex relationships and benefits more as δ increases.

1. Mask: the mask is applied to form the English word “kernel,” as illustrated in Figure 4a.
2. Noise: uncorrelated Gaussian white noise with mean 0 and variance 1.
3. Effective design signal: the effective design signal from the HCP working memory task is used with the contrast **C** = [1, −1, 0]^*T*^. As shown in Figure 4b with a bold line.
4. Added signal: the design signal from the working memory task is linearly added to each masked voxel with a fixed contrast and additional noise: [1, −1, 0]^*T*^+δ𝒩(0, 1), where 𝒩(0, 1) denotes a Gaussian distribution with mean 0 and variance 1. The parameter δ controls the magnitude of the deviation between the added signal and the effective design signal. In practice, a larger δ corresponds to greater inter-subject variability in neural activation. For this simulation, we vary δ∈[0, 0.5]. Two examples with δ = 0.1 and δ = 0.4 are shown in Figure 4b, c, respectively. In previous studies, δ was chosen to be 0.1 (Yang et al., 2020).
5. Signal strength: for the normalized added signal, the signal strength ρ = 0.03 is used to balance the noise and the added signal. In previous studies, ρ was chosen such that GLM produced an AUC with FPR < 0.1 between 0.035 and 0.05 (Yang et al., 2020), which in our cases correspond to ρ∈ (0.025 and 0.035).
6. Spatial smoothing: for two-dimensional problems, four spatially oriented filters are defined in Appendix 7.1. The smoothing filter is set to a full width at half maximum (FWHM) equal to 2 pixels, normalized to the pixel size, as shown in Figure 2a. The equally weighted summation of these filters is equivalent to a single FWHM = 2 pixels Gaussian filter, ensuring consistent levels of smoothing for both GLM and kernel methods.

Once the ground truth is defined and **Y**_simulated_ and **X**_eff_ are generated, we can run the nonlinear KCCA algorithm to obtain the activation pattern α, with the hyperparameters in the kernel mapping are chosen to maximize robustness against shuffling. For the simulated data, we will take the absolute value of the final results. The final activation pattern is then compared with the ground truth, and AUC_FPR < 0.1_ is computed for each subject. GLM with a single Gaussian smoothing filter with FWHM = 2 pixels is used for comparison.

In Figure 4, we present results using Gaussian noise and a fixed signal strength *s* = 0.03, while varying the deviation between **X**_add_ and **X**_eff_. In Figure 4b, the bold line represents the effective design signal **X**_eff_, and the light brown lines represent **X**_add_ for selected subjects with difference δ = 0.1. The differences are relatively small. In Figure 4c we display the same large difference with δ = 0.4. There are noticeable variations in detailed fluctuation strength between **X**_add_ and **X**_eff_, which are used to simulate the complexity and heterogeneity of neuronal responses. Figure 4d shows the AUC with an FPR smaller than 0.1. A larger δ corresponds to a greater deviation between **X**_add_ and **X**_eff_. In general, as deviation increases, nonlinear kernels consistently outperform linear kernels, with improvements exceeding 2%.

We make a few comments on this simulation. First, this simulation is highly simplified and does not incorporate many realistic brain structures. The primary purpose is to conduct controlled variable analysis to isolate specific effects. Similar simulations have been performed previously on 2D grids to test spatial constraints (Cordes et al., 2012; Yang et al., 2018). While we will compare more complex 3D simulations next, it is important to note that realistic simulations often reduce interpretability. Moreover, although this is a simplified simulation, the observed improvement—while modest—is statistically significant, as reflected in the effect of δ.

Second, we also examined other factors using controlled variables in this 2D simulation. Detailed comparisons are included in the ablation study in Section 5. Among various factors such as signal strength and noise type, we found that the deviation between **X**_add_ and **X**_eff_ produced the most significant differences between linear and nonlinear kernels.

In summary, we find that the deviation between **X**_add_ and **X**_eff_ is the primary factor influencing normalized accuracy. For real fMRI data, where the signal strength may fall within a typical range, but the actual signal deviates slightly from the design model, nonlinear kernels are expected to yield significant improvements.

### 3.2 fMRI simulation

**Figure 5:**
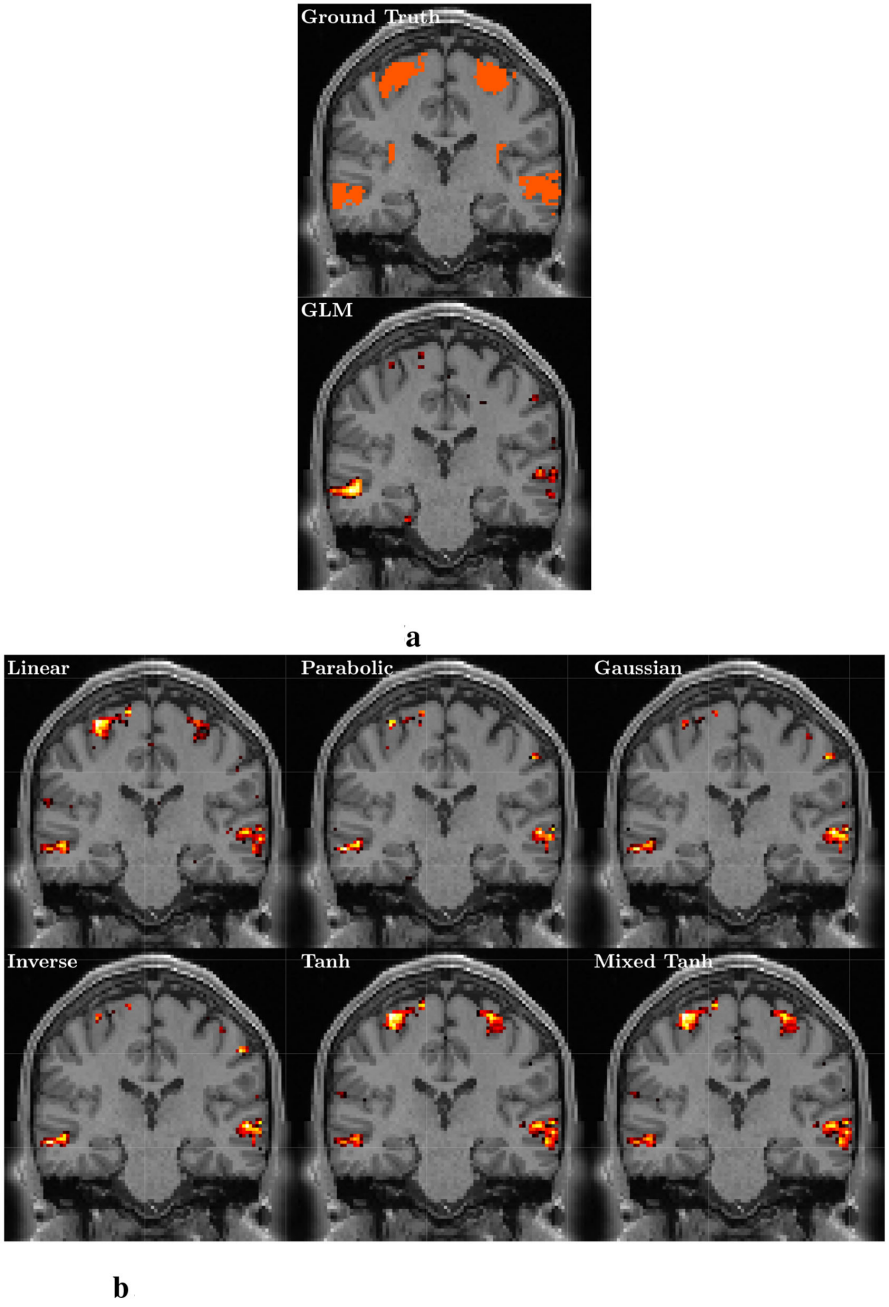
Ground truth and activation maps with *p* < 0.05 for one selected subject from the simulated fMRI. **(a)** Upper part: The ground truth, with the activation area highlighted in orange, along with the T1 image at the corresponding location for reference. **(a)** Lower part: The result from GLM, where the color indicates voxels with *p* < 0.05, and brighter colors represent larger |α| values. **(b)** Activation patterns for six different kernels using KCCA.

In this section, we generate another simulated dataset that more closely resembles the real task fMRI. The key components of the simulation are as follows:

1. Mask: the activation mask is selected for specific brain regions. We focus on 6 bilateral AAL regions (Tzourio-Mazoyer et al., 2002), including the anterior cingulate cortex, precentral gyrus, inferior frontal gyrus, insula, middle frontal gyrus, and middle temporal gyrus, which are treated as active. In total, ~10% of voxels in the brain (or around 19% of voxels in the gray matter) are activated. Let *M*∈ℜ^1 × *Q*^ be the mask which equals 1 for voxels belonging to chosen AAL regions and 0 otherwise (Tzourio-Mazoyer et al., 2002). An example of the activation is shown in Figure 5a.
2. Noise: the resampled resting-state is considered noise because it does not contain task-related information. Since the time dimension for the resting state in the HCP dataset is longer than that for the working memory task, we select the time frames between 301 and 690 to match the time dimension.
3. Effective design signal: Given three different event types: targets, non-targets, and lures contrasts. We use the contrast targets minus non-targets **C** = [1, −1, 0]^*T*^ to generate the effective design signal.
4. Added signal: the working memory-related signals are added to certain regions linearly. Similar to the previous 2D simulation, **X**_add_ is generated using a fixed contrast plus some random shift [1, −1, 0]^*T*^+0.1𝒩(0, 1).
5. Signal strength: along with the noise, activation mask, added signal, and effective design signal, the simulated data and ground truth are generated using Equation 6. The signal strength ρ is computed using Algorithm 1. Specifically, starting from ρ = 0.1 the algorithm will evaluate the performance from GLM method and compute the ROC curve. The value of ρ is adjusted—either increased or decreased—such that the AUC for FPR < 0.1 falls within the range of 0.035 to 0.05. This algorithm ensures that the signal strength is neither too high or not too low. Similar methods have been adopted in Yang et al. (2020).
6. Spatial smoothing: for GLM, a Gaussian smoothing filter with fixed FWHM = 4 mm is applied to the **Y**_simulated_. The correlation is computed between **X**_eff_ and **Y**_simulated_ after the smoothing. We ignore the sign for the correlation coefficient and normalize it by its maximum absolute value. For the kernel method, 7 SFs generated from FWHM = 4 mm are applied to the dataset. Detailed information about the smoothing kernels is provided in Appendix 7.1 and Figure 2b.

**Algorithm 1:** Algorithm to determine the signal strength ρ in the simulated data.

Generate **Y**_signal_, **Y**_noise_ and **X**_eff_. Start with ρ = 0.1.
while **True do**
Compute AUC based on GLM
if AUC_FPR < 0.1_ < 0.035 **then**
ρ = ρ+0.005
else **if** AUC_FPR < 0.1_>0.05 **then**
ρ = ρ−0.005
else
break
end **if**
end **while**

For this simulated data, the final result is evaluated based on |α|. The activation obtained from each kernel is then evaluated against the ground truth using the ROC curve. As a reference, the linear model with GLM is calculated with a single Gaussian smoothing with FWHM = 4 mm.

In Figure 5, we present an example from the simulated HCP data alongside the T1 image at the corresponding location. Figure 5a consists of two stacked images: the top image shows both the precentral gyrus (upper portion) and the mid-temporal region (lower portion), while the bottom image displays the GLM result, which reveals activation only in the mid-temporal region. Similar to GLM, Figure 5b shows that the parabolic, Gaussian, and inverse kernels also fail to capture activation in the precentral gyrus. In contrast, both structures are clearly detected using the hyperbolic tangent and mixed hyperbolic tangent kernels.

**Figure 6:**
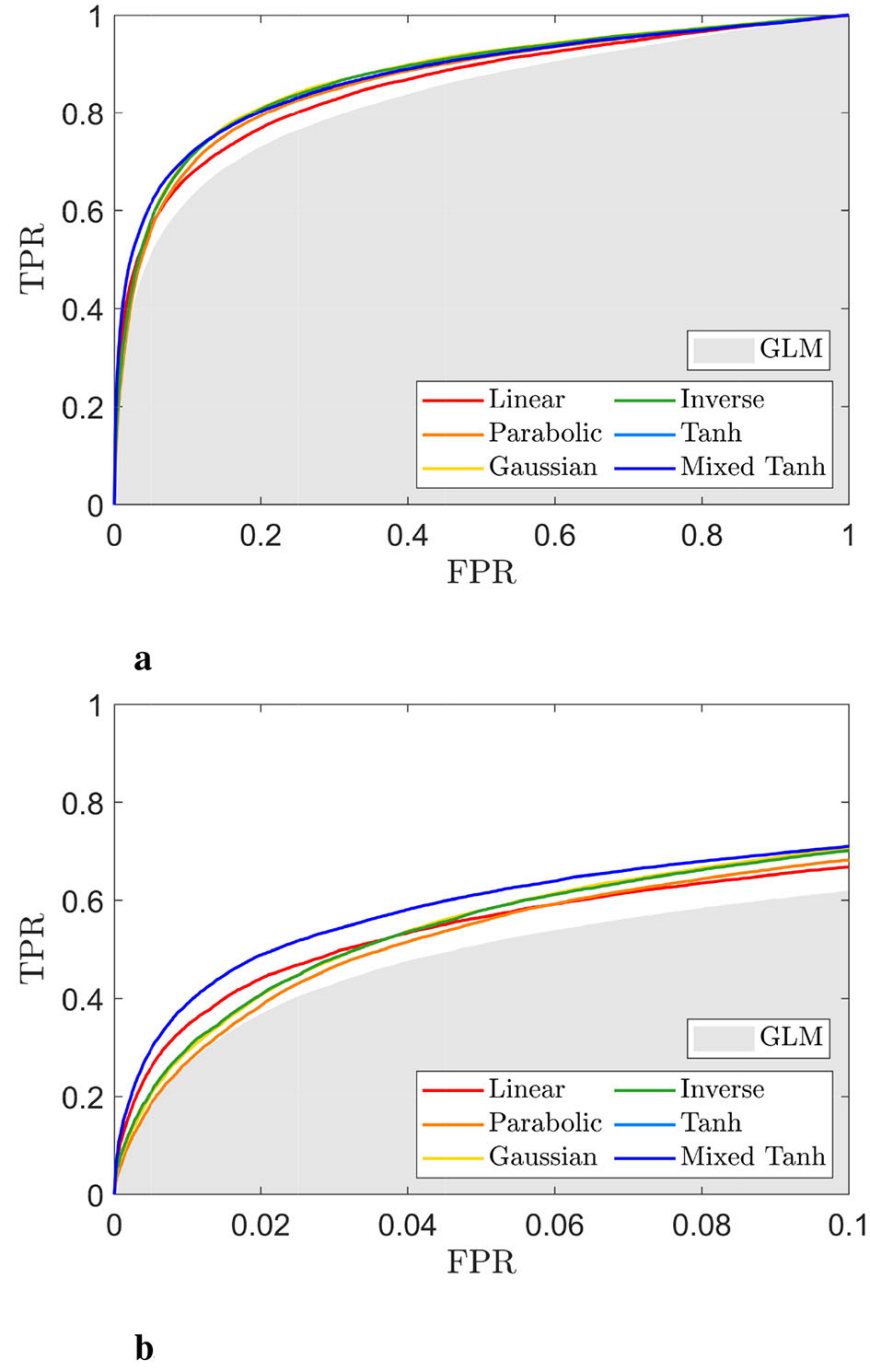
ROC curve for the selected subject from simulated fMRI data, with the activation pattern shown in Figure 5. **(a)** ROC curve for different kernels. The gray shading area indicates the AUC obtained from the GLM. **(b)** A focused view of **(a)** for FPR < 0.1. The mixed hyperbolic tangent kernel demonstrates the highest performance, achieving the largest AUC.

Figures 6a, b display the ROC curves for each kernel alongside the GLM. The AUC for FPR < 0.1 is 0.0528 for the linear kernel, 0.0510 for the parabolic kernel, 0.0531 for the Gaussian kernel, and 0.0530 for the inverse kernel. In comparison, both the hyperbolic tangent and mixed hyperbolic tangent kernels have AUC values of 0.0573, while the GLM, indicated by the gray shaded area, reaches only 0.0470. Therefore, for this specific subject, the accuracy normalized to GLM increases by ~22%.

The average performance for these 20 subjects is presented in Table 3, with the first column showing results normalized by GLM outcomes, focusing solely on the improvement rates. Parabolic, Gaussian, and inverse kernels do not perform well in this case, providing only a 5%–6% increase in accuracy. In contrast, the mixed hyperbolic tangent kernel demonstrates the maximum accuracy improvement, with a rate of 26.03%.

**Table 3:** Comparison of performance improvements among kernels across three different tasks.

**Kernel name**	**fMRI simulation**	**HCP**	**In-house scan**
Linear	21.24%	10.03%	20.10%
Parabolic	6.18%	10.01%	20.32%
Gaussian	5.61%	12.68%	22.14%
Inverse	5.33%	10.12%	22.35%
Tanh	19.94%	11.27%	23.41%
Mixed Tanh	**26.03%**	**24.27%**	**31.14%**

For the fMRI simulation, the ratio is defined as *r*_Truth_−1, while for the HCP and in-house scans, the ratio is defined as *r*_Gray_−1. For each task, the highest-performing method is shown in bold, and the second-best method is underlined.

We also compared the total AUC using the similar normalization techniques proposed in Equation 4. On average, the linear kernel and the mixed hyperbolic tangent kernel achieved accuracy improvements of 6.67 and 9.98%, respectively. Typically, this overall AUC gain is smaller than the gains observed at FPR < 0.1.

Additionally, we conducted the same simulations without taking the absolute value, and the results were nearly identical to those obtained with the absolute value. This suggests that the observed performance improvement is not attributable to the use of absolute value in this task.

## 4 Real fMRI data

### 4.1 HCP

For real fMRI data lacking a ground truth, we use the gray matter as a reference to evaluate final performance. We begin with the HCP dataset, from which the first 15 volumes of fMRI data are removed to prevent an unsaturated T1 signal. The computation method adheres to the procedures outlined in Section 2. The effective design signal generated by the contrast targets minus non-targets, denoted **C** = [1, −1, 0]^*T*^, is applied across all subjects. Similar to the analysis of simulated data, we assess the correlation coefficient between **X**_eff_ and **Y** after smoothing for each voxel, establishing this as the baseline model. We retain the sign of α or the correlation coefficient computed from GLM.

**Figure 7:**
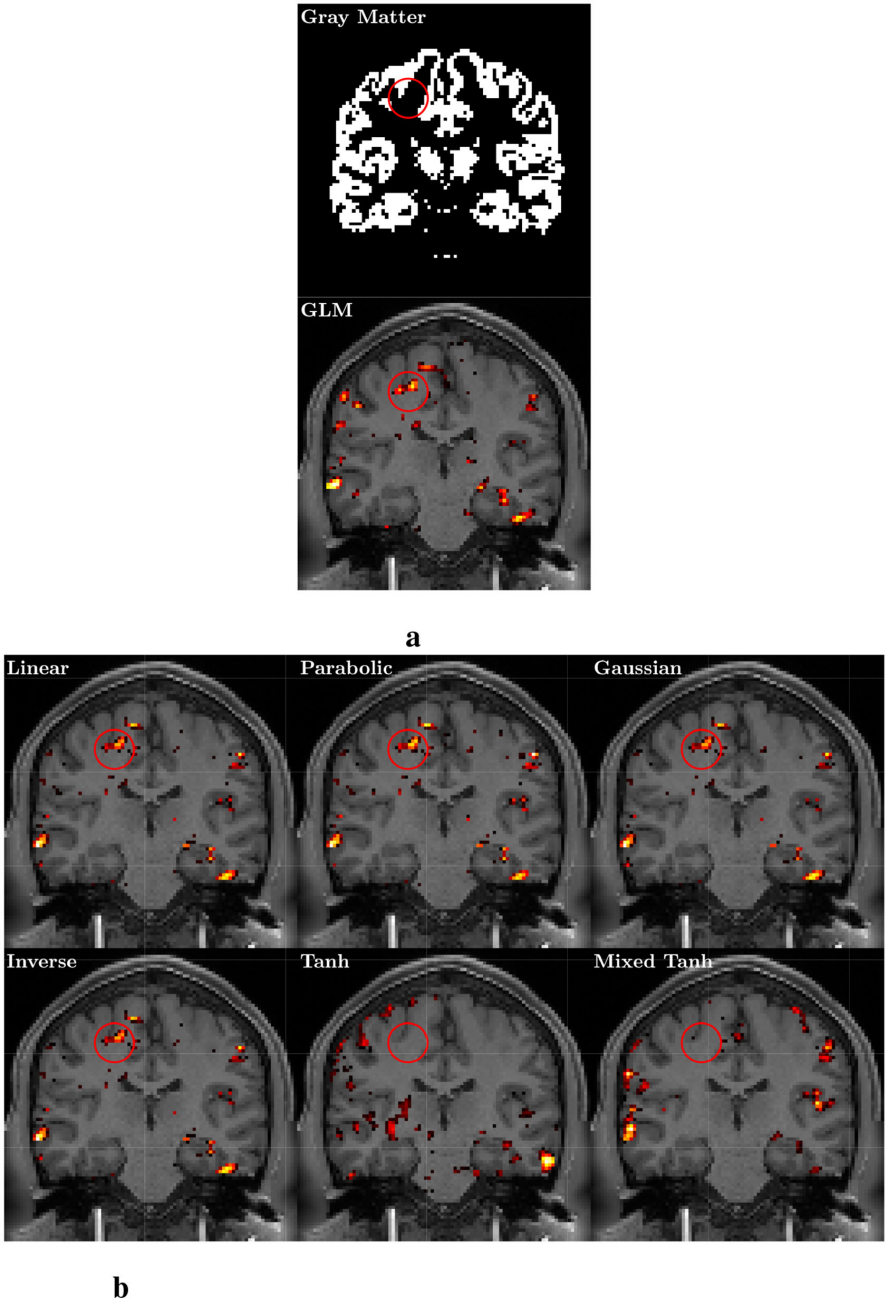
Gray matter and activation map with *p* < 0.05 for one selected subject from the real fMRI (HCP dataset). **(a)** The gray matter using segmentation probability larger than 0.5 (upper) and the result from GLM (lower). The color indicates voxels with *p* < 0.05, with a brighter color for larger α values. **(b)** Activation pattern for 6 different kernels using KCCA.

Figure 7 presents a selected example, with the upper part of Figure 7a showing that the gray matter serves as the reference. The results from the GLM are shown in the lower part of Figure 7a. For the kernel methods, both the fMRI data and the effective design signal are input into the nonlinear kernel method. Results for the six kernels from the back-reconstruction algorithms are displayed in Figure 7b. The activation maps derived from all six kernels generally show similar patterns to those observed in the GLM, although some subtle differences are noted. In the specific region marked by the red circle, most voxels belong to the white matter. The GLM, along with the linear, parabolic, Gaussian, and inverse kernels, reveals activation clusters within the circle, while the hyperbolic and mixed hyperbolic tangent kernels do not show this activation. However, the hyperbolic tangent kernel produces numerous small clusters in the white matter, which is not an ideal activation pattern. Conversely, the mixed hyperbolic tangent avoids activations in both undesired regions.

**Figure 8:**
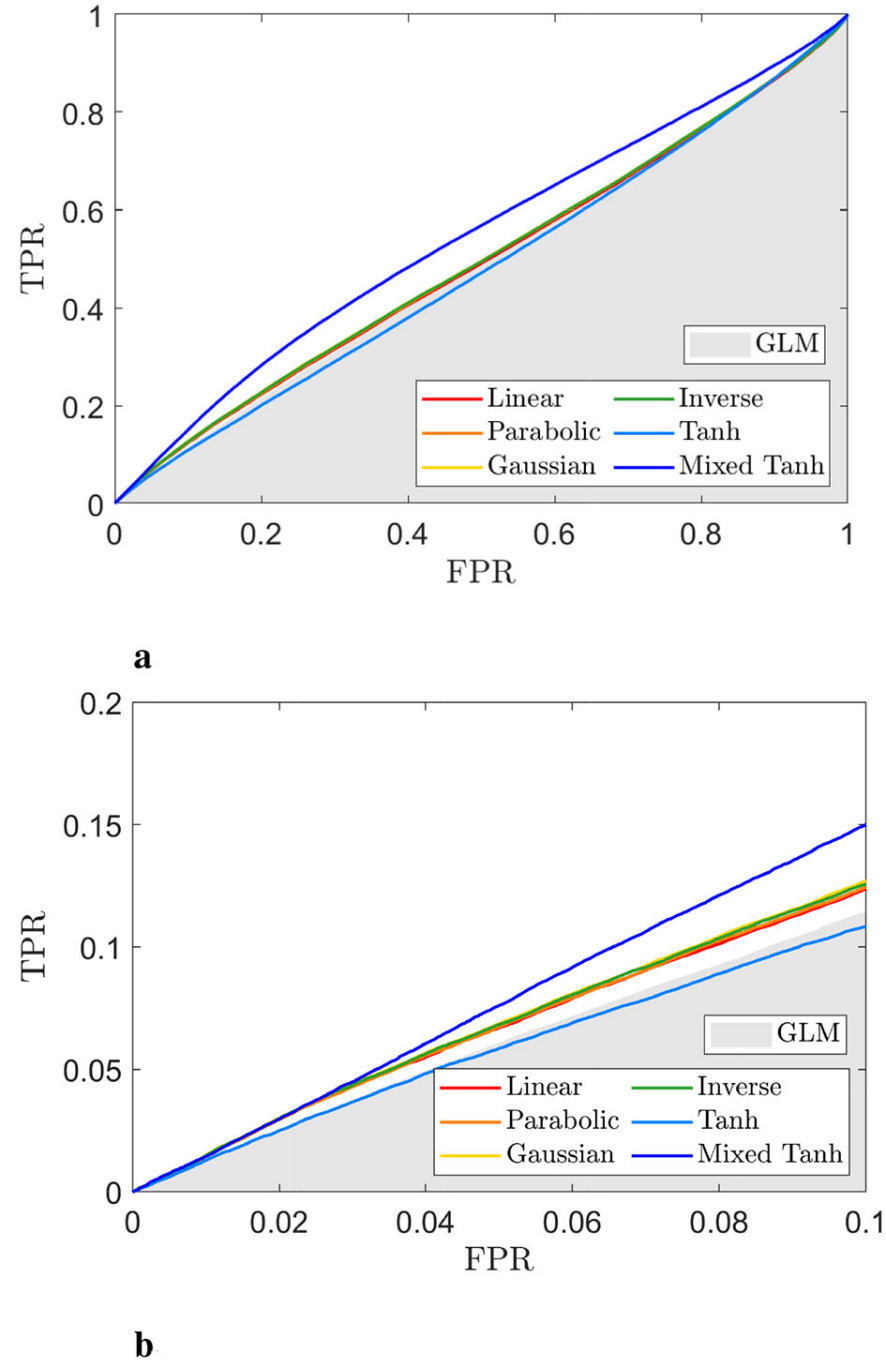
ROC curve for the selected subject from real fMRI data (HCP dataset), with the activation pattern shown in Figure 7. **(a)** ROC curves for different kernels, with the gray shaded area representing the AUC obtained from GLM. **(b)** A close-up view of **(a)** for FPR < 0.1. The hyperbolic tangent kernel shows poor performance, as indicated by scattered activations and its ROC curve. In contrast, the mixed hyperbolic tangent kernel demonstrates optimal performance in detecting activation within gray matter.

Figures 8a, b display ROC curves for the different kernels, using the gray matter as the ground truth. Since most voxels in the gray matter are inactive, the AUC is close to 0.5. When compared to the GLM, indicated by the gray shaded area, the mixed hyperbolic tangent kernel demonstrated the highest AUC and increased the number of activated voxels in the gray matter. In contrast, the hyperbolic tangent kernel shows less overlap with the gray matter, with portions of its curves falling within the gray shading area. Specifically, the AUC values with FPR < 0.1 for the six different kernels—linear, parabolic, Gaussian, inverse, hyperbolic tangent, and mixed hyperbolic tangent—are 6.55 × 10^−3^, 6.57 × 10^−3^, 6.70 × 10^−3^, 6.66 × 10^−3^, 5.72 × 10^−3^, and 7.56 × 10^−3^, respectively, whereas the GLM yields an AUC of 5.95 × 10^−3^. The mixed hyperbolic tangent kernel shows an increase of ~27%.

**Figure 9:**
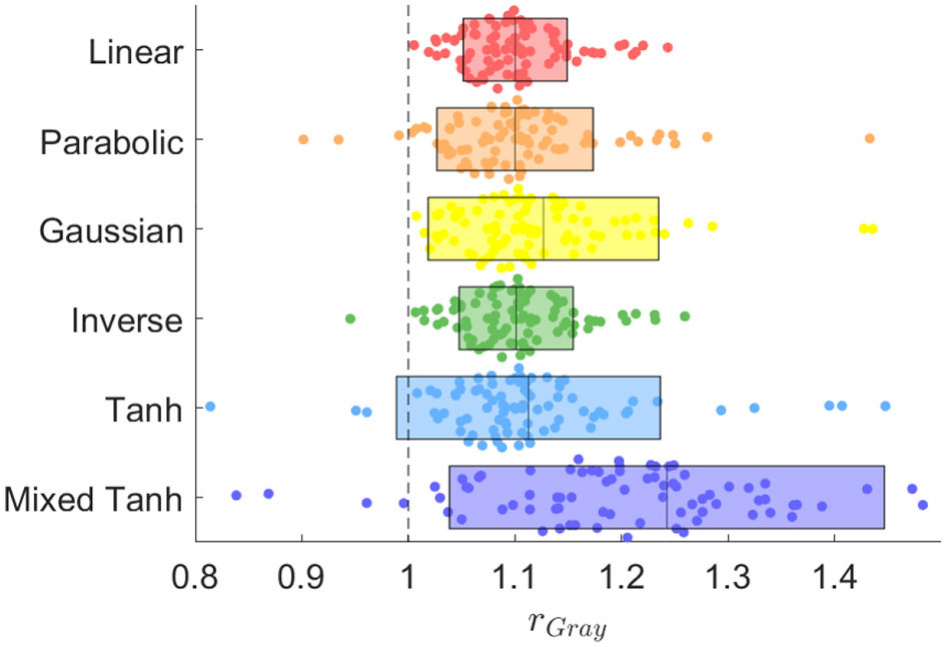
Swarm plot illustrating the AUC ratio for gray matter computed using different kernels in the HCP dataset, encompassing a total of 87 cases for each method. The rectangle represents the mean and variance for each method, while the black dashed line at *r*_Gray_ = 1 serves as the reference result from the GLM method.

In Figure 9, we present a swarm plot for each method applied to 87 subjects. Here, *r*_Gray_ measures the relative gray matter overlap of activation using GLM as a reference. The exact values are provided in the second column in Table 3. On average, the mixed hyperbolic tangent method outperforms the others, demonstrating a 24.27% increase compared to GLM. In contrast, the linear kernel exhibits a 10.03% increase. Moreover, the total AUC increase for activation within gray matter using the linear and the mixed hyperbolic tangent kernel is 0.55% and 5.34%, respectively. These results indicate that our nonlinear kernels can effectively avoid activations in undesired regions when compared to traditional methods.

### 4.2 In-house scan

We utilized in-house scans as an additional real fMRI dataset to evaluate the performance of nonlinear kernel methods. This dataset contains 16 subjects, including eight with amnestic mild cognitive impairment (aMCI) and eight normal controls. Trained professionals diagnosed aMCI based on Petersen Criteria (Petersen et al., 2001). Each subject completed an episodic memory task using images selected from human faces or various pictures (Jin et al., 2012). The tasks included four different events: instruction, control, encoding, and recognition, resulting in a contrast vector **X**∈ℜ^*T*×4^. After normalization to MNI, the fMRI data had dimensions 91 × 109 × 91 with *T* = 288. The first 10 seconds of data were removed to avoid an unsaturated T1 signal. For each subject and each task, we employed two different contrasts: encoding minus control (E-C) with contrast vector **C** = [0, −1, 1, 0]^*T*^ and recognition minus control (R-C) with **C** = [0, −1, 0, 1]^*T*^. Therefore, combining two tasks and two contrasts, results in an effective total of 64 subjects (16 subjects × 2 tasks × 2 contrasts).

**Figure 10:**
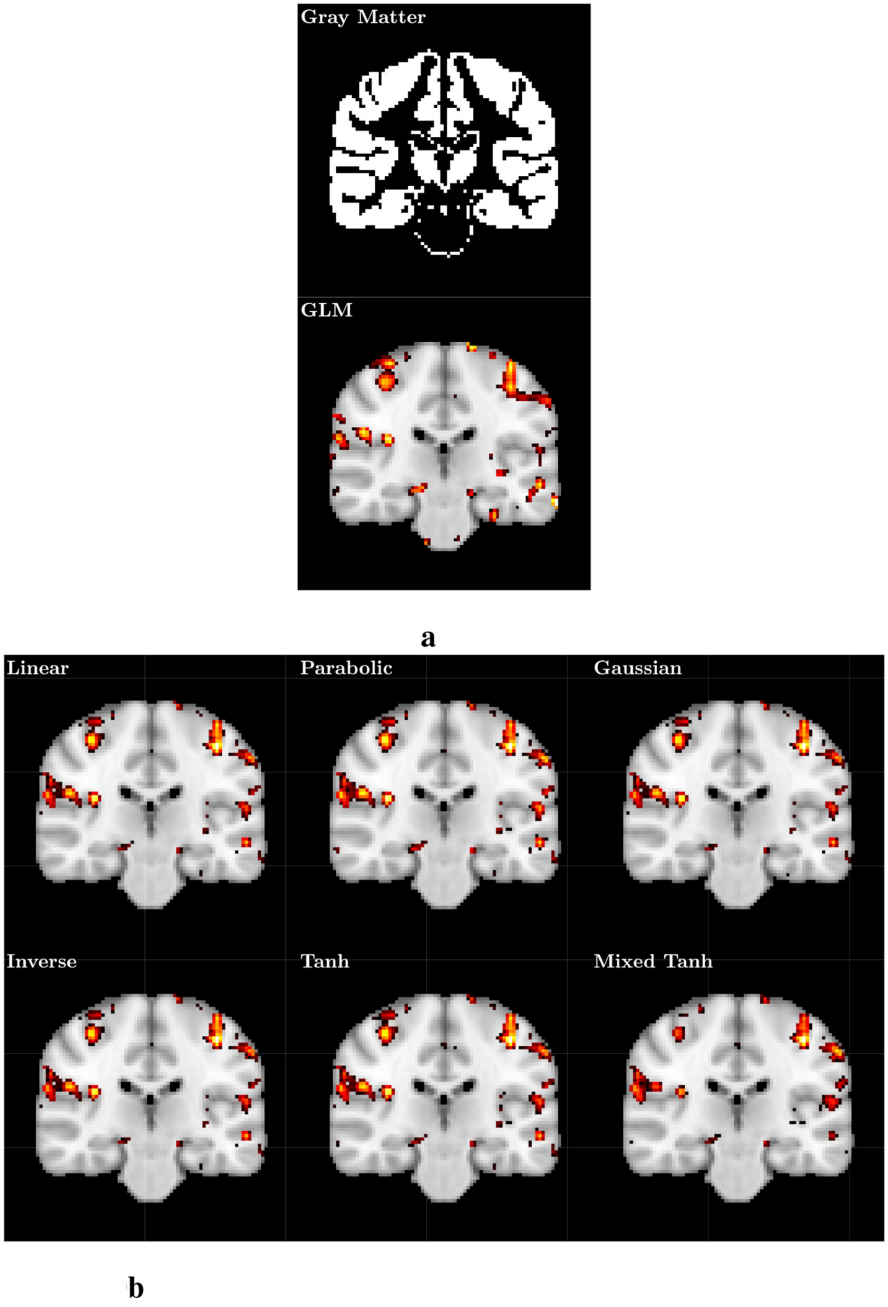
Gray matter and activation map with *p* < 0.05 for one selected subject from real fMRI (in-house dataset). **(a)** upper portion: The gray matter was obtained using segmentation probabilities >0.5. **(a)** lower portion: Results from GLM, where the color indicates voxels with *p* < 0.05, with brighter colors representing larger values of α. **(b)** Activation pattern for 6 different kernels using KCCA.

**Figure 11:**
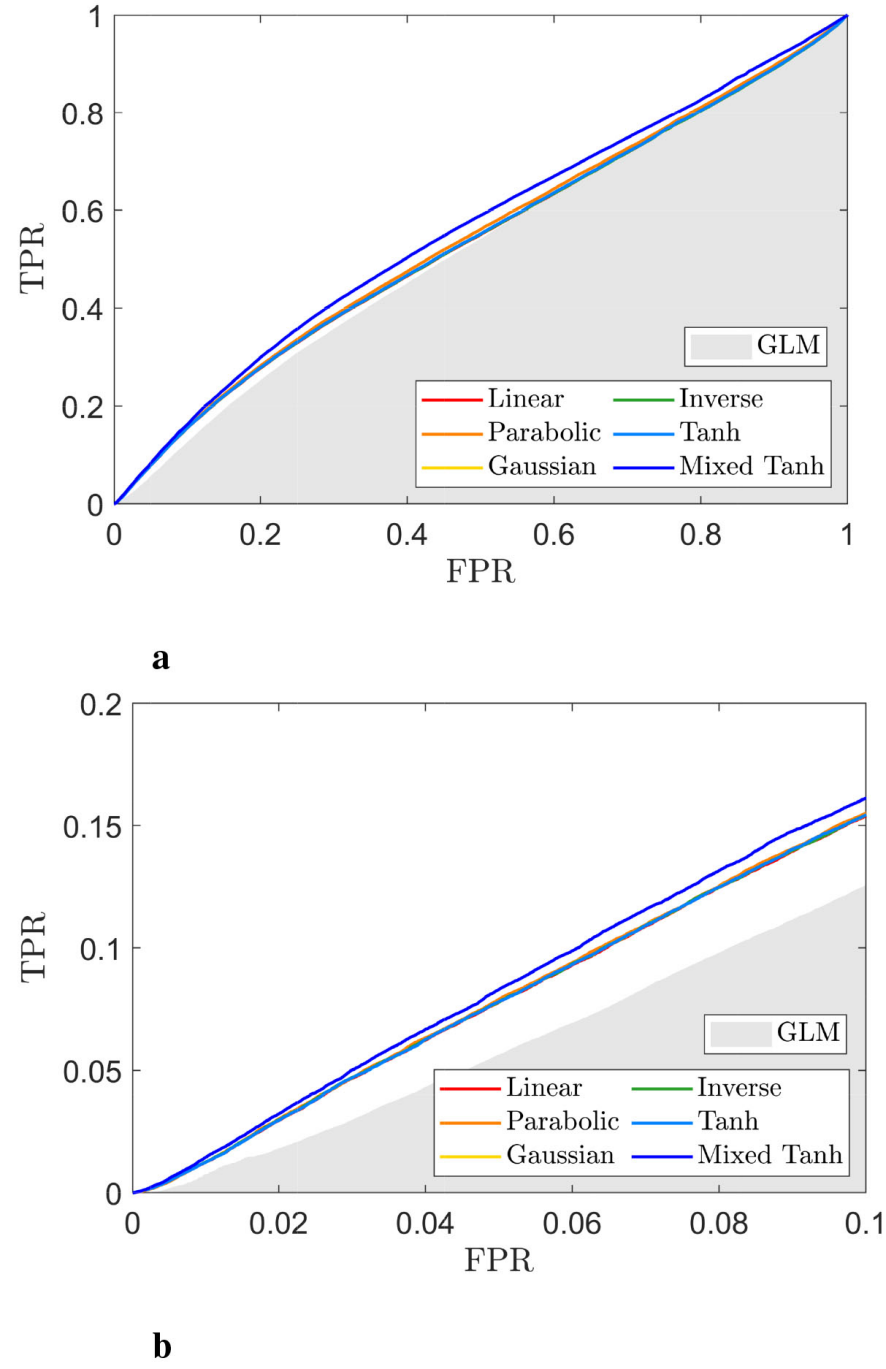
ROC curve for the selected subject from real fMRI data (in-house dataset), with the activation pattern shown in Figure 10. **(A)** ROC curves for different kernels, with the gray shaded area representing the AUC obtained from GLM. **(B)** A focused view of **(A)** for FPR < 0.1. The hyperbolic tangent kernel shows poor performance, as indicated by scattered activations and its corresponding ROC curve. In contrast, the mixed hyperbolic tangent kernel demonstrates optimal performance in detecting activation within gray matter.

The nonlinear kernels and GLM methods used in this analysis are the same as those described for the HCP dataset in Section 4.1. An example from one subject performing the faces task with the E-C contrast is shown in Figures 10, 11.

For the activation pattern, the lower panel of Figure 10a presents the GLM results as a reference, showing some activations at the bottom. In contrast, the upper panel of Figure 10b shows that these activations disappear when using kernel methods. Across the different kernels, Figure 10b indicates that their performance is similar, as further supported by Figure 11a. Notably, the performance of the mixed hyperbolic tangent kernel appears to be highest. The exact AUC values with FPR < 0.1 for linear, parabolic, Gaussian, inverse, hyperbolic tangent and mixed hyperbolic tangent kernels are 7.70 × 10^−3^, 7.78 × 10^−3^, 7.73 × 10^−3^, 7.73 × 10^−3^, 7.73 × 10^−3^ and 8.19 × 10^−3^, respectively. In comparison, GLM yields an AUC of 5.79 × 10^−3^. Notably, for this dataset, the kernel methods demonstrate greater accuracy compared to GLM. For example, the current subject shows an ~41% increase in accuracy for the mixed hyperbolic tangent kernel, normalized against GLM results.

**Figure 12:**
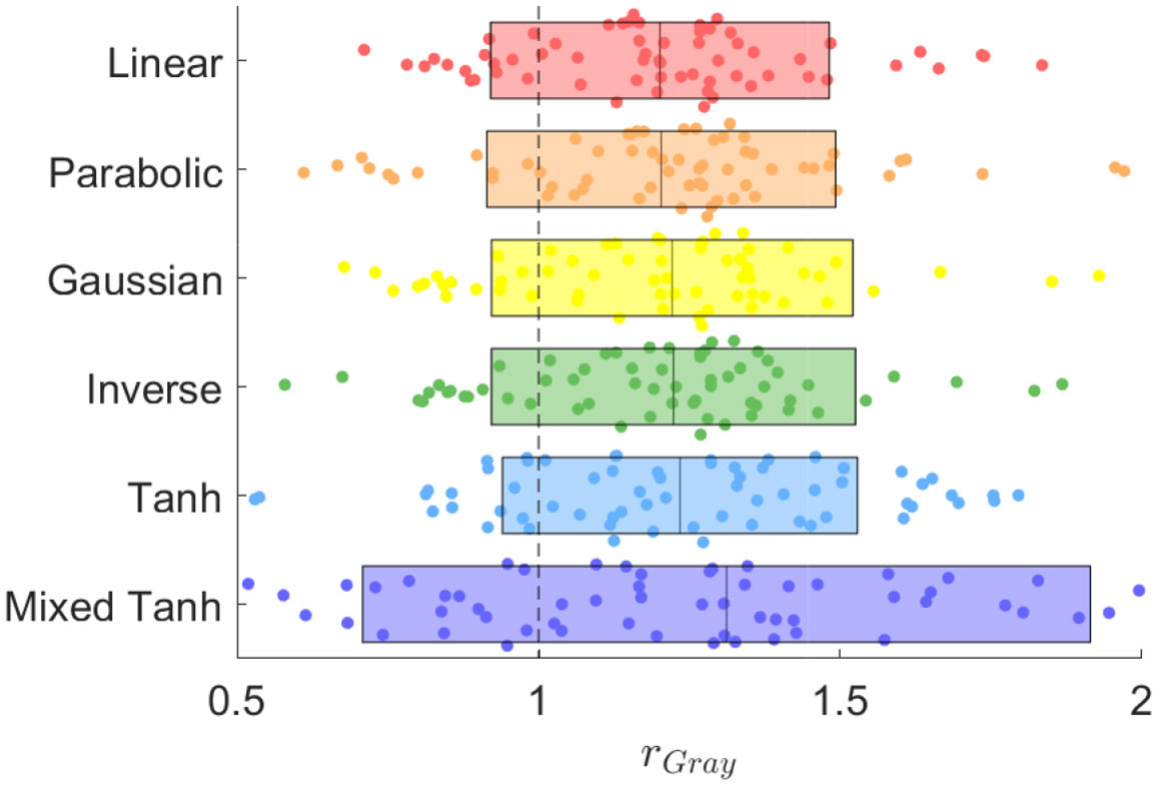
Swarm plot illustrating the AUC ratio for gray matter computed using different kernels in the in-house scans dataset, with a total of 64 dots for each method derived from 16 subjects, two tasks and two contrasts per subject. The rectangle represents the mean and variance for each method, and the black dashed line at *r*_Gray_ = 1 serves as the reference result from GLM.

Figure 12 presents the group-level analysis for all 16 subjects across two tasks and two contrasts. In Figure 12, we display a swarm plot for all cases, along with the mean and variance for each kernel method. The specific improvement ratio for AUC with FPR < 0.1 shown in Table 3, and the total AUC increase for the linear and the mixed hyperbolic tangent kernel is 4.47 and 2.93%, respectively. One notable observation is that in some instances, the kernels demonstrate more significant improvements in gray matter overlapping activation compared to the HCP dataset. Upon analyzing the cases with a high enhancement rate, we find that this is usually due to these cases having relatively large head movement or the correlation coefficient from GLM being concentrated in the negative regions. Despite these factors, the group-level analysis confirms that the mixed hyperbolic tangent kernel consistently outperforms the other kernels.

## 5 Ablation study

To further validate the differences between linear and nonlinear kernels, we use the same activation mask as in Figure 4a and perform control variable analysis across the three tasks listed below:

**Task 1**. Signal Strength Effect: Gaussian white noise is used, with no deviation between the added signal and the design signal (δ = 0). The signal strength is varied with ρ∈[0.02, 0.05].
**Task 2**. Autoregressive Noise (AR1) Effect: With δ = 0 and ρ = 0.03, the added noise follows an autoregressive model: (_*Y*_noise_)*t*_ = ϕ(_*Y*_noise_)*t*−1_+𝒩(0, 1). In previous studies involving 3T fMRI data, the autocorrelation parameter ϕ is typically chosen between 0.1 and 0.4 (Cordes and Nandy, 2007; Li, 2014). In our study, we vary ϕ from 0 to 0.4.
**Task 3**. Nonlinear Effect: When ϕ = 0, ρ = 0.03, and δ∈[0, 0.5], this task is the same as the simulation setup shown in Figure 4.

**Figure 13:**
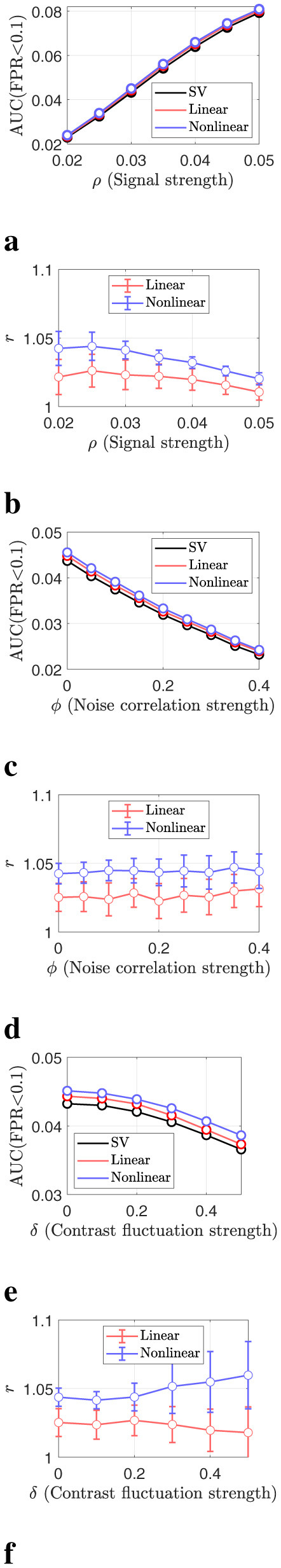
Group-level analysis of the 2D toy model across three different conditions. **(a, b)** Original and normalized AUC values vs. signal strength. **(c, d)** Original and normalized AUC values vs. noise correlation. **(e, f)** Original and normalized AUC values vs. signal deviation. Control variable analysis reveals that only signal deviation produces significant improvement in performance for the nonlinear kernel.

Figure 13 summarizes the results from the three tasks, including both original and normalized AUC values. In Figures 13a, b, we present results for Task 1, which examines the effect of signal strength. Figure 13a shows the AUC (with FPR < 0.1) for three methods: GLM, linear kernel, and nonlinear kernel. For the nonlinear kernel, the mixed hyperbolic tangent is used as the representative method. Each dot represents the average performance over 200 simulations. Across all methods, the AUC increases with signal strength, indicating a positive correlation between signal strength and detection accuracy.

Figure 13b shows the normalized accuracy difference between the nonlinear and linear kernels, with GLM used for normalization. The improvement from nonlinear kernels decreases as signal strength increases. This trend is expected, as higher signal strength corresponds to an easier detection problem, where linear methods are sufficient. In conclusion, while nonlinear kernels offer statistically significant improvements at low signal strengths, the magnitude of the improvement is modest, ~1%.

For Task 2, Figures 13c, d show the effects of varying noise correlation strength. Previous studies have reported typical autocorrelation values between 0.1 and 0.4 for 3T fMRI data (Cordes and Nandy, 2007; Li, 2014). In Figure 13c, we observe that for a fixed signal strength and added signal type, the AUC decreases with increasing noise correlation for all methods (GLM, linear kernel, and nonlinear kernel). For GLM, this decline is likely due to its assumption of independent Gaussian noise being violated. Figure 13d shows the normalized accuracy difference between the nonlinear and linear kernels. Unlike Task 1, there is no clear pattern between noise correlation and performance difference, suggesting that the benefit of nonlinear kernels is not strongly influenced by temporal autocorrelation in the noise.

For Task 3, Figures 13e, f present results with Gaussian noise (ϕ = 0), fixed signal strength (ρ = 0.03), and increasing deviation between the added signal **X**_add_ and the effective design signal **X**_eff_. Figure 13e shows that AUC generally decreases as the deviation increases. Despite this, nonlinear kernels consistently outperform linear kernels. Figure 13f shows the normalized accuracy difference, which increases with larger deviations. In this case, the improvement exceeds 2%, highlighting the robustness of nonlinear kernels when modeling more complex relationships.

In summary, among the three factors—signal strength, noise correlation, and deviation between **X**_add_ and **X**_eff_—the deviation appears to be the primary determinant of normalized accuracy improvement. For real fMRI data, where signal strength may fall within a reasonable range but the actual neural response may differ slightly from the design model, nonlinear kernels are expected to offer significant performance gains.

## 6 Discussion

### 6.1 Nonlinear kernel

#### 6.1.1 The advantage of nonlinear kernels

Traditional linear regression relies on two primary assumptions: the existence of a strictly linear relationship and the presence of independent Gaussian noise. When these assumptions are violated, the validity of the linear model is compromised, necessitating the use of nonlinear methods.

In fMRI correlation analysis, linear models assume that the activation signal aligns with the effective design signal, which is produced using a fixed hypothetical neural response. However, this assumption may not hold true due to the complicated HRF response in real brain activation (Glover, 1999; Lindquist et al., 2009). Consequently, a sole focus on linear relationships may be insufficient for detecting activation. To illustrate this point, we conducted a simulation by controlling variables in a 2D toy model. Our findings reveal that even slight modifications to the temporal pattern, as depicted in Figures 13e, f, resulted in significant differences in normalized accuracy between linear and nonlinear methods.

Moreover, real fMRI data often exhibit noise that is more complex than independent Gaussian noise. One noise model adopted in previous studies is autoregressive noise (Cordes and Nandy, 2007; Li, 2014). Using the same 2D toy model as a reference, we observed that, under constant signal strength, the accuracy of the linear model (measured by the AUC with FPR < 0.1) decreases as noise correlation increases, which aligns with expectations. In this scenario, we did not observe any performance improvements for nonlinear models, as shown in Figures 13c, d. Conversely, low-frequency noise, stemming from hardware instabilities or residual movement effects, has also been considered in prior analyses (Lund et al., 2006). Introducing low-frequency noise into the data effectively modifies the true activation signal, as in our first scenario, suggesting that nonlinear models may be more broadly applicable under these noise conditions.

Spatial smoothing and kernel mapping can slightly alter the results. For example, in a linear simulation with ϕ = 0 and δ = 0 as shown in Figures 13a, b, the normalized accuracy indicates that the nonlinear kernel outperforms the linear model when the signal strength is low. However, this improvement is relatively minor (< 1%), particularly when compared to the significant performance variations observed across different signal strengths.

Two factors are not addressed in our results. First, instead of simulating multiple regions with distinct HRFs to generate varying effective design signals (Glover, 1999; Lindquist et al., 2009; Yang et al., 2020), we use the same effective design signals for each subject, which may not precisely match the added signal. While this approach sacrifices some spatial heterogeneity, it avoids artifacts introduced by smoothing filters that span multiple regions with different added signals. Moreover, our method more clearly demonstrates the advantages of nonlinear models. We also tested the same setup using various contrasts, specifically applying six different contrasts (as proposed in Yang et al., 2020) in Figure 4a while using a single contrast for testing. Under these conditions, we again observed improved performance with nonlinear kernels.

Second, rather than modifying the HRF (Glover, 1999; Lindquist et al., 2009; Yang et al., 2020), which involves multiple hyperparameters, we opted to adjust only the contrasts for simplicity. As illustrated in Figures 4b, c, this approach effectively modifies the temporal pattern.

#### 6.1.2 Kernel selection

Using different datasets, we observed that the mixed hyperbolic tangent kernel outperforms the linear kernel and provides better performance compared to commonly used kernels such as parabolic or Gaussian. While a rigorous mathematical explanation is difficult, our empirical understanding is as follows: (1) the mixed hyperbolic tangent kernel can reduce to a linear kernel in special cases, offering flexibility for both linear and nonlinear problems; (2) previous studies have shown that mixed kernels—combining multiple components—can yield better performance (Zhu et al., 2012); and (3) bounded kernels tend to be more resilient to noise than unbounded ones (Alam, 2014).

#### 6.1.3 Other nonlinear methods in fMRI analysis

One example is the Support Vector Machine (SVM), which typically appears in classification problems. A similar inverse mapping algorithm is used to obtain the sensitivity map (Rasmussen et al., 2011). To the best of our knowledge, this method has not been applied to activation analysis in task fMRI. The second example is Deep CCA (Yang et al., 2025), which has recently been adopted for nonlinear analysis in task fMRI. Similar to CCA with constraints, Deep CCA remains within the local family (Zhuang et al., 2017, 2020), while our method is part of the global family, enabling activation detection across the entire brain in a single step.

Another approach involves using deep learning-based models. One example is deep learning-based denoising for task fMRI (Yang et al., 2020). The main idea is that the correlation in gray matter exceeds that in white matter, which automatically bypasses the white matter activation issue. Another model gaining recent attention is the transformer, which integrates spatial and temporal convolutions (Kim et al., 2023). This technique usually requires many datasets and complex techniques, such as self-supervised learning, to avoid overfitting. However, our model remains useful for capturing nonlinear relationships from single subjects.

### 6.2 Back-reconstruction algorithm

Previous publications have highlighted the challenges associated with defining back-reconstruction algorithms that utilize nonlinear kernels (Yang et al., 2018). For linear kernels, the feature map is defined as $\varphi \left({\tilde{\text{Y}}}_{t}\right)=\left({Ỹ}_{t1},{Ỹ}_{t2}\cdots \;,{Ỹ}_{tP}\right)$, which does not mix different voxels, allowing for a direct definition of activation. In contrast, nonlinear kernels can lead to feature maps that evolve with a mixture of terms or an infinite number of components. For example, the parabolic kernel when *b*^2^ = 0 reduces to ${\left(K_{\text{Y}}\right)}_{ij}={\left(\overset{P}{\underset{x=1}{\sum }}{Ỹ}_{ix}{Ỹ}_{jx}\right)}^{2}$ with *P*^2^ terms, the corresponding feature map is defined as $\varphi \left({\tilde{\text{Y}}}_{t}\right)=\left({Ỹ}_{t1}^{2},{Ỹ}_{t2}^{2},\cdots \;,{Ỹ}_{tP}^{2},{Ỹ}_{t1}{Ỹ}_{t2},{Ỹ}_{t1}{Ỹ}_{t3},\cdots \;,{Ỹ}_{tp_{1}}{Ỹ}_{tp_{2}},\cdots \;\right)$ with *P* square terms and *P*(*P*−1) cross terms. To address this issue, we draw on concepts from computer vision to compute voxel importance. Several equivalent terms have been proposed, including CAM (Zhou et al., 2016; Selvaraju et al., 2017), sensitivity maps (Smilkov et al., 2017), and saliency maps (Simonyan et al., 2013). This concept was adopted as an expansion of the nonlinear kernel mapping as an effective way to extend the current algorithm to general types of nonlinear mappings.

Compared to previously published methods, our back-reconstruction algorithm also involves calculating the derivative from the kernel space to the original space. Both approaches include smoothing techniques—for example, the Gaussian smoothing used in CAM (Zhou et al., 2016; Selvaraju et al., 2017). The key difference is that, in our method, the derivative is computed solely from the kernel matrix *K*_**Y**_, without taking the derivative with respect to **v**_**Y**_. Excluding **v**_**Y**_ is necessary to maintain consistency with the original mapping from kernel space to voxel space in the special case of a linear kernel.

### 6.3 Additional comments on methods

#### 6.3.1 Comparison between SF and Gaussian filter

Univariate analyses, such as GLM, typically utilize a single filter, whereas kernel methods can accommodate more complex filters, such as the SFs proposed in this study. Previous studies employing SFs for linear KCCA have demonstrated advantages in reducing spatial blurring (Yang et al., 2018). To fully leverage the capabilities of KCCA, we applied SFs across all kernel-based approaches.

#### 6.3.2 Sign effect in activation analysis

A critical question is whether to retain the sign of α during activation analysis. In previous GLM studies (Friman et al., 2003), the sign is preserved to indicate that only signals positively correlated with the effective design signal are selected. Conversely, earlier local CCA studies (Zhuang et al., 2017) adjusted weights to ensure positive correlation, resulting in activation patterns that may include both positive and negative components.

In this study, α is treated as the temporal average of the changing rate between each voxel and the time series in the kernel space. Because the kernel-space signal can have both positive and negative components, determining whether to retain the sign is not straightforward. To address this issue, we consider the unique properties of the linear kernel and treat the sign as an extension of local correlation.

For the simulated data, we conducted an additional test using the signed equation without applying the absolute value. Similar to Figure 4, we used independent Gaussian noise with δ = 0, ϕ = 0, and ρ = 0.035. When applying the absolute value, the average AUC over 200 subjects at FPR < 0.1 for the linear kernel and the mixed hyperbolic tangent kernel is 0.0554 and 0.0561, with average normalized improvements of 2.21 and 3.58%, respectively. With the signed equation, the average AUC at FPR < 0.1 for the linear kernel and the mixed hyperbolic tangent kernel is 0.0638 and 0.0642, with average normalized improvements of 1.09 and 1.84%, respectively. Although using the signed equation increases the AUC, the difference between linear and nonlinear methods remains nearly unchanged.

Generally, we find that when the problem is more linear, using the signed equation improves the AUC. In these cases, kernel mapping usually ensures that all active voxels are mapped to the positive region, making additional absolute-value operations unnecessary. Applying the absolute value is more beneficial when there are large deviations between the added signal and the design signal.

In real data analysis, we retain the sign of α due to the nature of the BOLD signal. A negative correlation may indicate blood outflow rather than inflow, which allows KCCA to revert to the original linear KCCA formulation (Hardoon et al., 2004; Yang et al., 2018).

#### 6.3.3 Shuffling method

In this study, we propose two shuffling methods. The first is an extension of the shuffling technique used for local CCA (Zhuang et al., 2017), with the goal of preserving the spatial structure. We aim to ensure that there is no significant difference between voxels before and after the shuffling algorithm. The second shuffling method is designed to generate a null distribution. A similar approach was previously proposed to maximize the difference between task fMRI and resting-state fMRI (Yang et al., 2020).

#### 6.3.4 Volume-based and surface-based activation analysis

The current manuscript focuses on a volume-based approach, treating fMRI data as a 3D time series and applying smoothing based on real spatial coordinates. We acknowledge the existence of surface-based analysis, which incorporates structural information (e.g., FreeSurfer) (Fischl, 2012) and reduces activation outside the gray matter (Mejia et al., 2020; Brodoehl et al., 2020). Our approach takes a different perspective: even without incorporating anatomical constraints, the proposed method demonstrates an ability to reduce activation in non-gray matter regions.

### 6.4 Evaluation

#### 6.4.1 Metrics for real fMRI activation

For real fMRI data, where the ground truth is unknown, several evaluation strategies have been proposed. For example, Nandy and Cordes (2004) suggests a novel approach to generate ROC curves without relying on ground truth. In this work, we assume that BOLD activations occur primarily in gray matter (Logothetis and Wandell, 2004). The rationale includes: first, it has been shown that gray matter has three to four times the cerebral blood flow and volume compared to white matter (Gawryluk et al., 2014). Second, the BOLD signal in gray matter is three to six times larger than in white matter, with the latter also showing time delays or even negative correlations with stimulation (Schilling et al., 2023). The standard choice for HRF may not be suitable for white matter; as a result, this increases the rationale for treating white matter as false activation. Third, unlike surface-based methods, which require anatomical structures (Mejia et al., 2020; Brodoehl et al., 2020), or task denoising methods (Yang et al., 2020), our volume-based approach does not require such information, making gray matter suitable for independent testing. Fourth, this metric is consistent with earlier investigations that examined whether identified activations align with detailed gray matter profiles (Yang et al., 2018, 2025).

#### 6.4.2 Reduction of false positives

Our algorithms primarily focus on the AUC when the FPR is < 0.1. Similar methods have been adopted in Yang et al. (2018). Beyond this threshold, well-performing methods generally yield better overall performance for the total AUC, although the total AUC itself typically changes at a relatively small rate.

### 6.5 Limitations

#### 6.5.1 Losses of local information

A key limitation of our methodology lies in the kernel mapping: while it enables the extraction of global relationships in a single step, it sacrifices local information, making it difficult to define an exact activation threshold. For example, traditional methods often involve resampling resting-state data to construct a null distribution, based on the observation that locally measured correlations can differ significantly between task fMRI and resampled resting-state data. However, nonlinear KCCA involves several normalization steps, and large correlations can appear in the kernel space regardless of the dataset, making it difficult to compute the null distribution in the same manner. To address this, we use reshuffling of **v**_**Y**_ to create an approximate null distribution. This distribution may include large values for α_null_, necessitating the use of a higher p-value threshold.

#### 6.5.2 Overfitting

Global methods such as KCCA determine the activation pattern using more voxels than time points. As a result, KCCA will always find a correlation regardless of the input data or the nonlinear mapping. A traditional way to avoid overfitting is to add a regularization parameter γ during KCCA analysis (Hardoon et al., 2004).

Determining a suitable γ, as well as the nonlinear mapping, is another challenge. We propose using voxel shuffling robustness following Zhuang et al. (2017) to determine these hyperparameters. The assumption is that activation patterns that are more robust to voxel shuffling will have larger gray matter overlap. We also note that this is only an assumption and could be violated. Moreover, this shuffling could create a large computational burden, especially for nonlinear kernels, as discussed in detail later.

From a performance perspective, our methodology occasionally leads to unstable results with the nonlinear kernel method. Although it generally enhances the likelihood of detecting activation overlaying gray matter in most subjects, there are instances where its performance drops below the baseline. These instances often occur when the kernel produces activations that are more resistant to voxel shuffling but do not align well with gray matter. This is similar to traditional overfitting issues observed in machine learning studies. Future research could focus on developing more robust nonlinear methods to address these limitations.

#### 6.5.3 Computational speed

Another limitation of the nonlinear kernel is its increased computational time. The back-reconstruction step takes *O*(*TQ*) time, while the kernel mapping step requires *O*(*T*^2^*Q*). Linear kernels involve one hyperparameter, whereas the mixed hyperbolic tangent kernel involves four and requires mapping through two kernels. As a result, the mixed hyperbolic tangent kernel is 200 × 2/15 ~27 times slower than the linear kernel when processing data from each subject to the activation map.

The additional time consumption primarily comes from determining the hyperparameter at the subject level. Without anatomical information, one practical approach is to compute the correlation difference between task and resting-state fMRI (Yang et al., 2018). This method avoids multiple mappings from the original space to the kernel space. We tried this for the nonlinear kernel; however, due to strong overfitting in the model, we found that maximizing the difference was not sufficient to create a robust activation pattern.

### 6.6 Application

#### 6.6.1 Importance of a general inverse mapping method

The practical application of neuroscience is to build interpretation models with anatomically defined patterns. As a result, not only convolution from image to features but also the inverse mapping is desired. Commonly used inverse mapping algorithms in computer vision include CAM (Zhou et al., 2016; Selvaraju et al., 2017), sensitivity maps (Smilkov et al., 2017), and saliency maps (Simonyan et al., 2013); they are all, in some way, based on computing the gradient from the output with respect to the input image. Our method follows this approach, with some revisions to make the model fit the linear kernel result. We also note that this type of approach is not limited to KCCA or volume-based fMRI data analysis and could have potential for other techniques, such as kernel-based classification or surface-based approaches.

Apart from using the gradient, another way to define inverse mapping is to use a model to directly predict the activation based on a certain objective function. For example, KCCA can be used to maximize the difference between task and resting state (Yang et al., 2018), or deep learning can be used to maximize the difference between gray matter and white matter (Yang et al., 2020). Similar mathematical modeling appears in computer vision for adversarial attacks to maximize the difference (Goodfellow et al., 2014).

#### 6.6.2 Modulating data with HRF variability

One practical application of this method is its suitability for datasets with substantial HRF variability. Previous studies (Handwerker et al., 2004; West et al., 2019) have reported significant differences in HRF across subjects; for example, in West et al. (2019), some of the hyperparameters for HRF changed by nearly half across different age groups, with clear HRF variations across subjects. This suggests that a single HRF may not be suitable for a heterogeneous group. Our proposed nonlinear KCCA method has proven robust to these changes and produces better results under the same signal-to-noise ratio, which may be ideal for modeling HRF variability.

#### 6.6.3 Integration with HRF estimation or deconvolution

One possible extension of our framework is to integrate it with existing HRF estimation or deconvolution methods. In practice, HRF estimation aims to recover latent neural activity by modeling and removing the variability of the hemodynamic response across brain regions and subjects (Wu et al., 2021). This step could be applied before our nonlinear KCCA analysis, providing input time series that are already compensated for HRF variability. Alternatively, our nonlinear kernel mapping could be embedded into the HRF estimation pipeline itself, for example by applying kernel-based correlation analysis to the deconvolved signals to enhance sensitivity to subtle activations. Such integration may improve robustness in heterogeneous populations, where HRF shape differences are substantial, and could facilitate more accurate comparisons across different subjects or brain regions. Potential challenges include additional computational cost and the need to avoid overfitting.

## 7 Conclusion

In this study, we introduce a new methodology for detecting fMRI activation using nonlinear kernels and canonical correlation analysis. The main contributions of this study are twofold:

We introduce a novel back-reconstruction algorithm that maps from the kernel space to the original space. Validation of this method includes: (1) measuring the contribution of each voxel to the kernel, (2) ensuring that, in the case of the linear kernel, it aligns with previous publications, and (3) demonstrating improved performance across different datasets.

We present a comparison between linear and nonlinear methods under different conditions, with a particular focus on control variables in two-dimensional simulations. The results show that nonlinear kernels outperform linear ones when the added signal has a small variance relative to the effective design signal. In real fMRI data, individual differences in neural responses create a similar scenario, further supporting the advantage of nonlinear kernels over linear ones.

## Data Availability

The datasets presented in this study can be found in online repositories. The names of the repository/repositories and accession number(s) can be found in the article/Supplementary material.

## References

[B1] Akaho S. (2006). A kernel method for canonical correlation analysis. arXiv [Preprint]. 10.48550/arXiv.cs/0609071

[B2] Alam M. A. (2014). Kernel Choice for Unsupervised Kernel Methods. Hayama: Graduate University for Advanced Studies, SOKENDAI.

[B3] Ashburner J. (2009). Computational anatomy with the SPM software. Magn. Reson. Imaging 27, 1163–1174. 10.1016/j.mri.2009.01.006 19249168

[B4] Behzadi Y. Restom K. Liau J. Liu T. T. (2007). A component based noise correction method (COMPCOR) for bold and perfusion based fMRI. Neuroimage 37, 90–101. 10.1016/j.neuroimage.2007.04.042 17560126 PMC2214855

[B5] Bengio Y. Delalleau O. Roux N. L. Paiement J.-F. Vincent P. Ouimet M.. (2004). Learning eigenfunctions links spectral embedding and kernel PCA. Neural Comput. 16, 2197–2219. 10.1162/0899766041732396 15333211

[B6] Brodoehl S. Gaser C. Dahnke R. Witte O. W. Klingner C. M. (2020). Surface-based analysis increases the specificity of cortical activation patterns and connectivity results. Sci. Rep. 10:5737. 10.1038/s41598-020-62832-z 32235885 PMC7109138

[B7] Bullmore E. Long C. Suckling J. Fadili J. Calvert G. Zelaya F.. (2001). Colored noise and computational inference in neurophysiological (fMRI) time series analysis: resampling methods in time and wavelet domains. Hum. Brain Mapp. 12, 61–78. 10.1002/1097-0193(200102)12:2&lt;61::AID-HBM1004&gt;3.0.CO;2-W 11169871 PMC6871881

[B8] Chen B. Yang J. Jeon B. Zhang X. (2017). Kernel quaternion principal component analysis and its application in RGB-D object recognition. Neurocomputing 266, 293–303. 10.1016/j.neucom.2017.05.047

[B9] Chen J. E. Jahanian H. Glover G. H. (2017). Nuisance regression of high-frequency functional magnetic resonance imaging data: denoising can be noisy. Brain Connect. 7, 13–24. 10.1089/brain.2016.0441 27875902 PMC5312601

[B10] Cordes D. Jin M. Curran T. Nandy R. (2012). Optimizing the performance of local canonical correlation analysis in fMRI using spatial constraints. Human Brain Mapp. 33, 2611–2626. 10.1002/hbm.21388 23074078 PMC5551496

[B11] Cordes D. Nandy R. (2007). Independent component analysis in the presence of noise in fMRI. Magn. Reson. Imaging 25, 1237–1248. 10.1016/j.mri.2007.03.021 17509787

[B12] Erhardt E. B. Allen E. A. Wei Y. Eichele T. Calhoun V. D. (2012). Simtb, a simulation toolbox for fMRI data under a model of spatiotemporal separability. Neuroimage 59, 4160–4167. 10.1016/j.neuroimage.2011.11.088 22178299 PMC3690331

[B13] Esteban O. Markiewicz C. J. Blair R. W. Moodie C. A. Isik A. I. Erramuzpe A.. (2019). fMRIprep: a robust preprocessing pipeline for functional MRI. Nat. Methods 16, 111–116. 10.1038/s41592-018-0235-4 30532080 PMC6319393

[B14] Fasshauer G. E. (2007). Meshfree Approximation Methods with MATLAB. London: World Scientific Pub Co Inc. 10.1142/6437

[B15] Fischl B. (2012). Freesurfer. Neuroimage 62, 774–781. 10.1016/j.neuroimage.2012.01.021 22248573 PMC3685476

[B16] Frackowiak R. S. Friston K. J. Frith C. D. Dolan R. J. Price C. J. Zeki S.. (2004). Chapter 40 - analysis of fMRI time series: linear time-invariant models, event-related fMRI, and optimal experimental design. Hum. Brain Funct. 2004, 793–822. 10.1016/B978-012264841-0/50042-1

[B17] Friman O. Borga M. Lundberg P. Knutsson H. (2003). Adaptive analysis of fMRI data. NeuroImage 19, 837–845. 10.1016/S1053-8119(03)00077-6 12880812

[B18] Friman O. Cedefamn J. Lundberg P. Borga M. Knutsson H. (2001). Detection of neural activity in functional MRI using canonical correlation analysis. Magn. Reson. Med. 45, 323–330. 10.1002/1522-2594(200102)45:2&lt;323::AID-MRM1041&gt;3.0.CO;2-# 11180440

[B19] Gawryluk J. R. Mazerolle E. L. D'Arcy R. C. (2014). Does functional MRI detect activation in white matter? A review of emerging evidence, issues, and future directions. Front. Neurosci. 8:239. 10.3389/fnins.2014.00239 25152709 PMC4125856

[B20] Glasser M. F. Sotiropoulos S. N. Wilson J. A. Coalson T. S. Fischl B. Andersson J. L.. (2013). The minimal preprocessing pipelines for the human connectome project. Neuroimage 80, 105–124. 10.1016/j.neuroimage.2013.04.127 23668970 PMC3720813

[B21] Glover G. H. (1999). Deconvolution of impulse response in event-related bold fMRI1. Neuroimage 9, 416–429. 10.1006/nimg.1998.0419 10191170

[B22] Goodfellow I. J. Shlens J. Szegedy C. (2014). Explaining and harnessing adversarial examples. arXiv [Preprint]. arXiv:1412.6572. 10.48550/arXiv.1412.6572

[B23] Han C. Yang Z. Zhuang X. Cordes D. (2024). “Nonlinear kernel-based fMRI activation detection,” in International Society for Magnetic Resonance in Medicine (Singapore).

[B24] Handwerker D. A. Ollinger J. M. D'Esposito M. (2004). Variation of bold hemodynamic responses across subjects and brain regions and their effects on statistical analyses. Neuroimage 21, 1639–1651. 10.1016/j.neuroimage.2003.11.029 15050587

[B25] Hardoon D. R. Szedmak S. Shawe-Taylor J. (2004). Canonical correlation analysis: an overview with application to learning methods. Neural Comput. 16, 2639–2664. 10.1162/0899766042321814 15516276

[B26] Jin M. Pelak V. S. Curran T. Nandy R. R. Cordes D. (2012). A preliminary study of functional abnormalities in AMCI subjects during different episodic memory tasks. Magn. Reson. Imaging 30, 459–470. 10.1016/j.mri.2011.12.014 22387024 PMC3327830

[B27] Karanikolas G. V. Giannakis G. B. Slavakis K. Leahy R. M. (2016). “Multi-kernel based nonlinear models for connectivity identification of brain networks,” in 2016 IEEE International Conference on Acoustics, Speech and Signal Processing (ICASSP) (Shanghai: IEEE), 6315–6319. 10.1109/ICASSP.2016.7472892

[B28] Kim P. Kwon J. Joo S. Bae S. Lee D. Jung Y.. (2023). “Swift: Swin 4d fMRI transformer,” in Advances in Neural Information Processing Systems, Vol. 36 (New Orleans, LA), 42015–42037.

[B29] Lai P. L. Fyfe C. (2000). Kernel and nonlinear canonical correlation analysis. Int. J. Neural Syst. 10, 365–377. 10.1142/S012906570000034X 11195936

[B30] Laird A. R. Rogers B. P. Meyerand M. E. (2004). Comparison of Fourier and wavelet resampling methods. Magn. Reson. Med. 51, 418–422. 10.1002/mrm.10671 14755671

[B31] Langs G. Menze B. H. Lashkari D. Golland P. (2011). Detecting stable distributed patterns of brain activation using GINI contrast. NeuroImage 56, 497–507. 10.1016/j.neuroimage.2010.07.074 20709176 PMC3960973

[B32] Li X. (2014). Functional Magnetic Resonance Imaging Processing. Berlin: Springer. 10.1007/978-3-642-39106-4

[B33] Lindquist M. A. Loh J. M. Atlas L. Y. Wager T. D. (2009). Modeling the hemodynamic response function in fMRI: efficiency, bias and mis-modeling. Neuroimage 45, S187–S198. 10.1016/j.neuroimage.2008.10.065 19084070 PMC3318970

[B34] Logothetis N. K. Wandell B. A. (2004). Interpreting the bold signal. Annu. Rev. Physiol. 66, 735–769. 10.1146/annurev.physiol.66.082602.092845 14977420

[B35] Lopez-Paz D. Sra S. Smola A. Ghahramani Z. Schölkopf B. (2014). “Randomized nonlinear component analysis,” in International Conference on Machine Learning (Beijing: PMLR), 1359–1367.

[B36] Lund T. E. Madsen K. H. Sidaros K. Luo W.-L. Nichols T. E. (2006). Non-white noise in fMRI: does modelling have an impact? Neuroimage 29, 54–66. 10.1016/j.neuroimage.2005.07.005 16099175

[B37] Mejia A. F. Yue Y. R. Bolin D. Lindgren F. Lindquist M. A. (2020). A Bayesian general linear modeling approach to cortical surface fMRI data analysis. J. Am. Stat. Assoc. 501–520. 10.1080/01621459.2019.1611582 33060871 PMC7556728

[B38] Melzer T. Reiter M. Bischof H. (2001). “Nonlinear feature extraction using generalized canonical correlation analysis,” in In International Conference on Artificial Neural Networks (Cham: Springer), 353–360. 10.1007/3-540-44668-0_50

[B39] Nandy R. R. Cordes D. (2003). Novel roc-type method for testing the efficiency of multivariate statistical methods in fMRI. Magn. Resonance Med. 49, 1152–1162. 10.1002/mrm.10469 12768594

[B40] Nandy R. R. Cordes D. (2004). New approaches to receiver operating characteristic methods in functional magnetic resonance imaging with real data using repeated trials. Magn. Resonance Med. 52, 1424–1431. 10.1002/mrm.20263 15562482

[B41] Ni Y. Chu C. Saunders C. J. Ashburner J. (2008). “Kernel methods for fMRI pattern prediction,” in 2008 IEEE International Joint Conference on Neural Networks (IEEE World Congress on Computational Intelligence) (Hong Kong: IEEE), 692–697. 10.1109/IJCNN.2008.4633870

[B42] Petersen R. C. Doody R. Kurz A. Mohs R. C. Morris J. C. Rabins P. V.. (2001). Current concepts in mild cognitive impairment. Arch. Neurol. 58, 1985–1992. 10.1001/archneur.58.12.1985 11735772

[B43] Poline J. Holmes A. Worsley K. Friston K. (1997). “Statistical inference and the theory of random fields,” in SPM Short Course Notes (Berkeley), 1–27.

[B44] Rasmussen P. M. Madsen K. H. Lund T. E. Hansen L. K. (2011). Visualization of nonlinear kernel models in neuroimaging by sensitivity maps. NeuroImage 55, 1120–1131. 10.1016/j.neuroimage.2010.12.035 21168511

[B45] Salazar J. Z. Kwakkel J. H. Witvliet M. (2024). Evaluating the choice of radial basis functions in multiobjective optimal control applications. Environ. Model. Softw. 171:105889. 10.1016/j.envsoft.2023.105889

[B46] Schilling K. G. Li M. Rheault F. Gao Y. Cai L. Zhao Y.. (2023). Whole-brain, gray, and white matter time-locked functional signal changes with simple tasks and model-free analysis. Proc. Natl. Acad. Sci. 120:e2219666120. 10.1073/pnas.2219666120 37824529 PMC10589709

[B47] Selvaraju R. R. Cogswell M. Das A. Vedantam R. Parikh D. Batra D.. (2017). “Grad-cam: visual explanations from deep networks via gradient-based localization,” in Proceedings of the IEEE International Conference on Computer Vision (Venice: IEEE), 618–626. 10.1109/ICCV.2017.74

[B48] Simonyan K. Vedaldi A. Zisserman A. (2013). Deep inside convolutional networks: Visualising image classification models and saliency maps. arXiv [Preprint]. arXiv:1312.6034. 10.48550/arXiv.1312.6034

[B49] Skudlarski P. Constable R. T. Gore J. C. (1999). Roc analysis of statistical methods used in functional MRI: individual subjects. Neuroimage 9, 311–329. 10.1006/nimg.1999.0402 10075901

[B50] Smilkov D. Thorat N. Kim B. Viégas F. Wattenberg M. (2017). Smoothgrad: removing noise by adding noise. arXiv [Preprint]. arXiv:1706.03825. 10.48550/arXiv.1706.03825

[B51] Tzourio-Mazoyer N. Landeau B. Papathanassiou D. Crivello F. Etard O. Delcroix N.. (2002). Automated anatomical labeling of activations in SPM using a macroscopic anatomical parcellation of the mni MRI single-subject brain. Neuroimage 15, 273–289. 10.1006/nimg.2001.0978 11771995

[B52] Uurtio V. Bhadra S. Rousu J. (2019). “Large-scale sparse kernel canonical correlation analysis,” in International Conference on Machine Learning (Long Beach, CA: PMLR), 6383–6391.

[B53] Van Essen D. C. Ugurbil K. Auerbach E. Barch D. Behrens T. E. Bucholz R.. (2012). The human connectome project: a data acquisition perspective. Neuroimage 62, 2222–2231. 10.1016/j.neuroimage.2012.02.018 22366334 PMC3606888

[B54] West K. L. Zuppichini M. D. Turner M. P. Sivakolundu D. K. Zhao Y. Abdelkarim D.. (2019). Bold hemodynamic response function changes significantly with healthy aging. NeuroImage 188, 198–207. 10.1016/j.neuroimage.2018.12.012 30529628 PMC6450381

[B55] Worsley K. J. (1995). Estimating the number of peaks in a random field using the hadwiger characteristic of excursion sets, with applications to medical images. Ann. Stat. 23, 640–669. 10.1214/aos/1176324540

[B56] Wu G.-R. Colenbier N. Van Den Bossche S. Clauw K. Johri A. Tandon M.. (2021). RSHRF: a toolbox for resting-state HRF estimation and deconvolution. NeuroImage 244:118591. 10.1016/j.neuroimage.2021.118591 34560269

[B57] Yakupov R. Lei J. Hoffmann M. B. Speck O. (2017). False fMRI activation after motion correction. Hum. Brain Mapping 38, 4497–4510. 10.1002/hbm.23677 28580597 PMC5553448

[B58] Yang Z. Zhuang X. Lowe M. Cordes D. (2022). “A global spatially-adaptive method for task fMRI activation analysis with the advantage of alleviated spatial blurring,” in International Society for Magnetic Resonance in Medicine (London).

[B59] Yang Z. Zhuang X. Lowe M. J. Cordes D. (2025). A deep neural network for adaptive spatial smoothing of task fMRI data. Front. Neuroimaging 4:1554769. 10.3389/fnimg.2025.1554769 40365169 PMC12070436

[B60] Yang Z. Zhuang X. Sreenivasan K. Mishra V. Curran T. Byrd R.. (2018). 3D spatially-adaptive canonical correlation analysis: local and global methods. NeuroImage 169, 240–255. 10.1016/j.neuroimage.2017.12.025 29248697 PMC5856611

[B61] Yang Z. Zhuang X. Sreenivasan K. Mishra V. Curran T. Cordes D.. (2020). A robust deep neural network for denoising task-based fMRI data: an application to working memory and episodic memory. Med. Image Anal. 60:101622. 10.1016/j.media.2019.101622 31811979 PMC6980789

[B62] Zhang Y. Kimberg D. Y. Coslett H. B. Schwartz M. F. Wang Z. (2014). Multivariate lesion-symptom mapping using support vector regression. Hum. Brain Mapp. 35, 5861–5876. 10.1002/hbm.22590 25044213 PMC4213345

[B63] Zhou B. Khosla A. Lapedriza A. Oliva A. Torralba A. (2016). “Learning deep features for discriminative localization,” in Proceedings of the IEEE Conference on Computer Vision and Pattern Recognition (Las Vegas, NV: IEEE), 2921–2929. 10.1109/CVPR.2016.319

[B64] Zhu X. Huang Z. Shen H. T. Cheng J. Xu C. (2012). Dimensionality reduction by mixed kernel canonical correlation analysis. Pattern Recognit. 45, 3003–3016. 10.1016/j.patcog.2012.02.007

[B65] Zhuang X. Yang Z. Cordes D. (2020). A technical review of canonical correlation analysis for neuroscience applications. Hum. Brain Mapp. 41, 3807–3833. 10.1002/hbm.25090 32592530 PMC7416047

[B66] Zhuang X. Yang Z. Curran T. Byrd R. Nandy R. Cordes D.. (2017). A family of locally constrained CCA models for detecting activation patterns in fMRI. NeuroImage 149, 63–84. 10.1016/j.neuroimage.2016.12.081 28041980 PMC5493994

