# Supplementary material for: Nonlinear kernel-based fMRI activation detection
Source: Front Neuroimaging. 2025 Sep 10;4:1649749. doi: 10.3389/fnimg.2025.1649749 (PMC12457110; doi:10.3389/fnimg.2025.1649749)
Supplement: Supplementary file 1 [file Presentation_1.pdf]

Supplementary material for "Nonlinear kernel-based fMRI activation detection"

Chendi Han[1], Zhengshi Yang[1], Xiaowei Zhuang[1], and Dietmar Cordes[1,2,*]

[1]Cleveland Clinic Lou Ruvo Center for Brain Health, Las Vegas, NV, United States
[2]Department of Psychology and Neuroscience, University of Colorado, Boulder, CO, United States
[*]Corresponding author. Email: cordesd@ccf.org

7 Appendix

7.1 Steerable filters

7.1.1 Two dimensional SF

Suppose G_{iso} is chosen as a Gaussian function with FWHM equal to half of the Gaussian filter F_{orig} used in GLM. We define [1]

$$
\begin{aligned}
G_i(\mathbf{x}) &= (1 - G_{\text{iso}}(\mathbf{x}))\left[\left(\hat{\mathbf{n}}_i^T \hat{\mathbf{x}}\right) - \frac{1}{6}\right], \\
\hat{\mathbf{n}}_1 &= \begin{pmatrix} 1 \\ 0 \end{pmatrix},\ \hat{\mathbf{n}}_2 = \begin{pmatrix} a \\ b \end{pmatrix},\ \hat{\mathbf{n}}_3 = \begin{pmatrix} -a \\ b \end{pmatrix}, \qquad (1)\\
a &= \frac{1}{2},\ b = \frac{\sqrt{3}}{2},
\end{aligned}
$$

and

$$F_{\text{iso}}(\mathbf{x}) = G_{\text{iso}}(\mathbf{x}),\ F_i(\mathbf{x}) = G_i(\mathbf{x})F_{\text{orig}}(\mathbf{x}) \text{ for } i = 1, 2, 3. \qquad (2)$$

Now F_{iso} and F_i can be treated as the SF. As both G_{iso} and F_{orig} decay for large $|\mathbf{x}|$, we can expand them to a finite grid as shown in Figure 2a.

For simplicity of analysis, a matrix form of these filters can be introduced easily. Define $\mathbf{A} \in \Re^{Q\times P}$ with

$$\mathbf{A} = [\mathbf{A}^{(0)},\ \mathbf{A}^{(1)},\ \mathbf{A}^{(2)},\ \mathbf{A}^{(3)}], \qquad (3)$$

where each $\mathbf{A}^{(i)}$ characterize one SF corresponding to F_{iso} and $F_{1,2,3}$ respectively. The matrix element in $\mathbf{A}_{ij}$ measures the contribution for ith voxel to jth voxel after transformation. We observe that when $\mathbf{x}$ changes to $-\mathbf{x}$, G_i and G_{iso} do not change. Then, each $\mathbf{A}^{(i)}$ is a symmetric matrix.

7.1.2 Three dimensional SF

Similarly, in 3D, we introduce G_{iso} which is selected as a Gaussian function with FWHM equal to half of the Gaussian filter F_{orig} used in GLM.

$$\begin{aligned}
G_i(\mathbf{x}) &= (1 - G_{\text{iso}}(\mathbf{x})) \left[\left(\hat{\mathbf{n}}_i^T \hat{\mathbf{x}} \right) - \frac{1}{6} \right], \\
\hat{\mathbf{n}}_1 &= \begin{pmatrix} a \\ 0 \\ b \end{pmatrix}, \ \hat{\mathbf{n}}_2 = \begin{pmatrix} -a \\ 0 \\ b \end{pmatrix}, \ \hat{\mathbf{n}}_3 = \begin{pmatrix} b \\ a \\ 0 \end{pmatrix}, \\
\hat{\mathbf{n}}_4 &= \begin{pmatrix} a \\ 0 \\ b \end{pmatrix}, \ \hat{\mathbf{n}}_5 = \begin{pmatrix} -a \\ 0 \\ b \end{pmatrix}, \ \hat{\mathbf{n}}_6 = \begin{pmatrix} b \\ a \\ 0 \end{pmatrix}, \\
a &= \frac{2}{\sqrt{10 + 2\sqrt{5}}}, \ b = \frac{1 + \sqrt{5}}{\sqrt{10 + 2\sqrt{5}}},
\end{aligned} \tag{4}$$

and

$$F_{\text{iso}}(\mathbf{x}) = G_{\text{iso}}(\mathbf{x}), \ F_i(\mathbf{x}) = G_i(\mathbf{x}) F_{\text{orig}}(\mathbf{x}) \text{ for } i = 1, \cdots, 6. \tag{5}$$

We can transform those filters to a matrix form $\mathbf{A}$ and decompose it to $\mathbf{A}^{(0,\cdots,6)}$, with each matrix being symmetric.

7.2 Chain rule for Eq.(3)

As K is defined by $\tilde{\mathbf{Y}}$, in order to compute the derivative in the original space we use the chain rule:

$$\begin{aligned}
\alpha_q &= \sum_{ij} \left(\frac{\partial K_{ij}}{\partial Y_{iq}} v_j \right) \\
&= \sum_{ij} \sum_{p} \left(\frac{\partial K_{ij}}{\partial \tilde{Y}_{ip}} \frac{\partial \tilde{Y}_{ip}}{\partial Y_{iq}} v_j \right) \\
&= \sum_{ij} \sum_{p} \left(\frac{\partial K_{ij}}{\partial \tilde{Y}_{ip}} \frac{\partial \sum_x Y_{ix} A_{xp}}{\partial Y_{iq}} v_j \right) \\
&= \sum_{ij} \sum_{p} \left(\frac{\partial K_{ij}}{\partial \tilde{Y}_{ip}} A_{qp} v_j \right).
\end{aligned} \tag{6}$$

For the first term in the bracket, $\tilde{\boldsymbol{\alpha}} \in \Re^{P \times 1}$ is easy to introduce in the filter space. Therefore, $\mathbf{Y}$ and $\boldsymbol{\alpha}$ are defined in the original space, $\tilde{\mathbf{Y}}$ and $\tilde{\boldsymbol{\alpha}}$ are in the filter space, and K and $\mathbf{v}$ are in the kernel space.

So that

$$\boldsymbol{\alpha} = \mathbf{A} \tilde{\boldsymbol{\alpha}}, \tag{7}$$

with

$$\tilde{\boldsymbol{\alpha}} = \sum_{ij} \frac{\partial K_{ij}}{\partial \tilde{\mathbf{Y}}_i} v_j. \tag{8}$$

7.3 Special cases for linear kernel

Introduce two normalization factors to ensure that the matrix elements for different kernels are consistent.

$$\begin{aligned}\mathcal{N}_{\text{linear}} &= \frac{1}{T^2}\sum_i\sum_j\left|\sum_p \tilde{Y}_{ip}\tilde{Y}_{jp}\right|,\\ \mathcal{N}_{\text{quadratic}} &= \frac{1}{T^2}\sum_i\sum_j\left|\sum_p\left(\tilde{Y}_{ip}-\tilde{Y}_{jp}\right)^2\right|.\end{aligned} \tag{9}$$

Starting from Eq. (8)

$$\begin{aligned}\tilde{\alpha}_p &= \sum_{ij}\frac{\partial K_{ij}}{\partial \tilde{Y}_{ip}}v_j\\ &= \frac{1}{\mathcal{N}_{\text{linear}}}\sum_{ij}\frac{\partial \sum_x \tilde{Y}_{ix}\tilde{Y}_{jx}}{\partial \tilde{Y}_{ip}}v_j\\ &= \frac{1}{\mathcal{N}_{\text{linear}}}\sum_{ij}\left[\tilde{Y}_{jp}v_j+\delta_{ij}\tilde{Y}_{jp}v_j\right]\\ &= \frac{T+1}{\mathcal{N}_{\text{linear}}}\tilde{\mathbf{Y}}^T\mathbf{v}.\end{aligned} \tag{10}$$

Finally we obtain

$$\boldsymbol{\alpha} = \frac{T+1}{\mathcal{N}_{\text{linear}}}\mathbf{A}\tilde{\mathbf{Y}}^T\mathbf{v} = \frac{T+1}{\mathcal{N}_{\text{linear}}}\mathbf{A}\mathbf{A}^T\mathbf{Y}^T\mathbf{v}. \tag{11}$$

7.4 Matrix representation for general types of kernel

Because the kernel matrix is derived from fMRI data after applying the spatial transform matrix, it is straightforward to first derive $\tilde{\boldsymbol{\alpha}}$ and then use Eq. (7) to transform back:

7.4.1 Linear kernel

We define

$$(K_{\text{linear}})_{ij} = \frac{1}{\mathcal{N}_{\text{linear}}}\sum_p \tilde{Y}_{ip}\tilde{Y}_{jp}. \tag{12}$$

As shown in Section. 7.3 for linear kernel we have

$$\tilde{\boldsymbol{\alpha}} = \frac{T+1}{\mathcal{N}_{\text{linear}}}\tilde{\mathbf{Y}}^T\mathbf{v} = c\tilde{\mathbf{Y}}^T\mathbf{v}, \tag{13}$$

where c is some positive constant.

7.4.2 Parabolic kernel

We define

$$(K_{\text{Parabolic}})_{ij} = \left(\frac{1}{\mathcal{N}_{\text{linear}}}\sum_x \tilde{Y}_{ix}\tilde{Y}_{jx}+b^2\right)^2. \tag{14}$$

Using Eq. (8)

$$
\begin{aligned}
\tilde{\alpha}_p =& \sum_{ij} \frac{\partial (K_{\text{Parabolic}})_{ij}}{\partial \tilde{Y}_{ip}} v_j \\
=& \sum_{ij} v_j \frac{\partial}{\partial \tilde{Y}_{ip}} \left[((K_{\text{linear}})_{ij})^2 + 2b^2 (K_{\text{linear}})_{ij} + b^4 \right] \\
=& \sum_{ij} v_j \left[2(K_{\text{linear}})_{ij} \frac{1}{\mathcal{N}_{\text{linear}}} \left(\tilde{Y}_{jp} + \delta_{ij} \tilde{Y}_{ip} \right) + \frac{2b^2}{\mathcal{N}_{\text{linear}}} \left(\tilde{Y}_{jp} + \tilde{Y}_{ip} \delta_{ij} \right) \right] \\
=& c \left[\sum_{ij} (K_{\text{linear}})_{ij} \left(\tilde{Y}_{jp} v_j + \tilde{Y}_{ip} v_j \delta_{ij} \right) + b^2 \sum_j (1+T) \tilde{Y}_{jp} v_j \right].
\end{aligned} \tag{15}
$$

7.4.3 Gaussian kernel

We define

$$
(K_{\text{Gaussian}})_{ij} = \exp \left[-\frac{1}{\sigma^2} \frac{1}{\mathcal{N}_{\text{quadratic}}} \sum_x \left(\tilde{Y}_{ix} - \tilde{Y}_{jx} \right)^2 \right]. \tag{16}
$$

Using Eq. (8)

$$
\begin{aligned}
\tilde{\alpha}_p =& \sum_{ij} \frac{\partial (K_{\text{Gaussian}})_{ij}}{\partial \tilde{Y}_{ip}} v_j \\
=& \sum_{ij} \exp \left[-\frac{1}{\sigma^2} \sum_x \frac{1}{\mathcal{N}_{\text{quadratic}}} \left(\tilde{Y}_{ix} - \tilde{Y}_{jx} \right)^2 \right] v_j \left(-\frac{1}{\sigma^2} \frac{1}{\mathcal{N}_{\text{quadratic}}} \right) \frac{\partial}{\partial \tilde{Y}_{ip}} \left[\sum_x \left(\tilde{Y}_{ix} - \tilde{Y}_{jx} \right)^2 \right] \\
=& -\frac{1}{\sigma^2 \mathcal{N}_{\text{quadratic}}} \sum_{ij} (K_{\text{Gaussian}})_{ij} \frac{\partial}{\partial \tilde{Y}_{ip}} \left(\tilde{Y}_{ip}^2 - 2\tilde{Y}_{ip} \tilde{Y}_{jp} + 2\tilde{Y}_{jp}^2 \right) \\
=& -\frac{1}{\sigma^2 \mathcal{N}_{\text{quadratic}}} \sum_{ij} (K_{\text{Gaussian}})_{ij} \left(\tilde{Y}_{ip} - \tilde{Y}_{jp} \right) v_j \\
=& c \sum_j v_j \left[-\left(\sum_i (K_{\text{Gaussian}})_{ij} \tilde{Y}_{ip} \right) + \left(\sum_i (K_{\text{Gaussian}})_{ij} \right) \tilde{Y}_{jp} \right].
\end{aligned} \tag{17}
$$

7.4.4 Inverse square root kernel

We define

$$
(K_{\text{Inverse}})_{ij} = 1 / \sqrt{\sum_x \frac{1}{\mathcal{N}_{\text{quadratic}}} (\tilde{Y}_{ix} - \tilde{Y}_{jx})^2 + b^2}. \tag{18}
$$

Using Eq. (8)

$$
\begin{aligned}
\tilde{\alpha}_p =& \sum_{ij} \frac{\partial (K_{\text{Inverse}})_{ij}}{\partial \tilde{Y}_{ip}} v_j \\
=& \sum_{ij} -\frac{(K_{\text{Inverse}})_{ij}^3}{2} \frac{1}{\mathcal{N}_{\text{quadratic}}} \frac{\partial [(\tilde{Y}_{ip} - \tilde{Y}_{jp})^2 + b^2]}{\partial \tilde{Y}_{ip}} v_j \\
=& \sum_{ij} -\frac{(K_{\text{Inverse}})_{ij}^3}{2} \frac{1}{\mathcal{N}_{\text{quadratic}}} \frac{\partial}{\partial \tilde{Y}_{ip}} \left(\tilde{Y}_{ip}^2 - 2\tilde{Y}_{ip}\tilde{Y}_{jp} + \tilde{Y}_{jp}^2 \right) v_j \\
=& c \sum_{ij} -(K_{\text{Inverse}})_{ij}^3 \left(\tilde{Y}_{ip} - \tilde{Y}_{jp} \right) v_j .
\end{aligned}
\tag{19}
$$

7.4.5 Hyperbolic tangent kernel

We define

$$
(K_{\text{Tanh}})_{ij} = \tanh \left(b \sum_x \frac{1}{\mathcal{N}_{\text{linear}}} \tilde{Y}_{ix} \tilde{Y}_{jx} + c \right) . \tag{20}
$$

Using Eq. (8)

$$
\begin{aligned}
\tilde{\alpha}_p =& \sum_{ij} \frac{\partial (K_{\text{Tanh}})_{ij}}{\partial \tilde{Y}_{ip}} v_j \\
=& \sum_{ij} \left[1 - (K_{\text{Tanh}})_{ij}^2 \right] \frac{b}{\mathcal{N}_{\text{linear}}} \frac{\partial (\tilde{Y}_{ix}\tilde{Y}_{jx})}{\partial \tilde{Y}_{ip}} v_j \\
=& \frac{b}{N_{\text{linear}}} \sum_{i,j} (1 - (K_{\text{Tanh}})_{ij}^2)(\tilde{Y}_{jp} + \delta_{ij}\tilde{Y}_{ip}) v_j \\
=& c \left[\sum_{ij} (1 - (K_{\text{Tanh}})_{ij}^2) \tilde{Y}_{jp} v_j + \sum_i (1 - (K_{\text{Tanh}})_{ii}^2) \tilde{Y}_{ip} v_i \right] .
\end{aligned}
\tag{21}
$$

7.4.6 Mixed hyperbolic tangent kernel

We define

$$
(K_{\text{Mixed Tanh}})_{ij} = \tanh \left[b_1 \frac{1}{\mathcal{N}_{\text{linear}}} \sum_x \tilde{Y}_{ix} \tilde{Y}_{jx} + b_2 \frac{1}{\mathcal{N}_{\text{quadratic}}} \sum_x \left(\tilde{Y}_{ix} - \tilde{Y}_{jx} \right)^2 + c \right] . \tag{22}
$$

Using Eq. (8)

$$
\begin{aligned}
\tilde{\alpha}_p =& \sum_{ij} \frac{\partial (K_{\text{Mixed Tanh}})_{ij}}{\partial \tilde{Y}_{ip}} v_j \\
=& \sum_{ij} \left[1 - (K_{\text{Mixed Tanh}})_{ij}^2 \right] \left[\frac{b_1}{\mathcal{N}_{\text{linear}}} \left(\tilde{Y}_{jp} v_j + \tilde{Y}_{ip} v_j \delta_{ij} \right) + \frac{b_2}{\mathcal{N}_{\text{quadratic}}} \left(2\tilde{Y}_{ip} - 2\tilde{Y}_{jp} \right) v_j \right] .
\end{aligned}
\tag{23}
$$

Expand the bracket. In total, we obtain 4 terms.

$$\begin{aligned}\tilde{\alpha}_p = &\sum_{ij} \frac{b_1}{\mathcal{N}_{\text{linear}}}(1-(K_{\text{Mixed Tanh}})^2_{ij})\tilde{Y}_{jp}v_j \\ &+ \sum_{i} \frac{b_1}{\mathcal{N}_{\text{linear}}}(1-(K_{\text{Mixed Tanh}})^2_{ii})\tilde{Y}_{ip}v_i \\ &+ \sum_{ij} \frac{2b_2}{\mathcal{N}_{\text{quadratic}}}(1-(K_{\text{Mixed Tanh}})^2_{ij})\tilde{Y}_{ip}v_j \\ &- \sum_{ij} \frac{2b_2}{\mathcal{N}_{\text{quadratic}}}(1-(K_{\text{Mixed Tanh}})^2_{ij})\tilde{Y}_{jp}v_j.\end{aligned} \tag{24}$$

References

[1] G. H. Granlund and H. Knutsson, *Signal processing for computer vision*. Springer Science & Business Media, 2013.
